# Structural and Biochemical Insights into the Mechanism of Action of the Clinical USP1 Inhibitor, KSQ-4279

**DOI:** 10.1021/acs.jmedchem.4c01184

**Published:** 2024-08-27

**Authors:** Martin Luke Rennie, Mehmet Gundogdu, Connor Arkinson, Steven Liness, Sheelagh Frame, Helen Walden

**Affiliations:** † School of Molecular Biosciences, College of Medical Veterinary and Life Sciences, University of Glasgow, Glasgow G12 8QQ, U.K.; ‡ Ubiquigent Ltd, Dundee University Incubator, James Lindsay Place, Dundee DD1 5JJ, U.K.

## Abstract

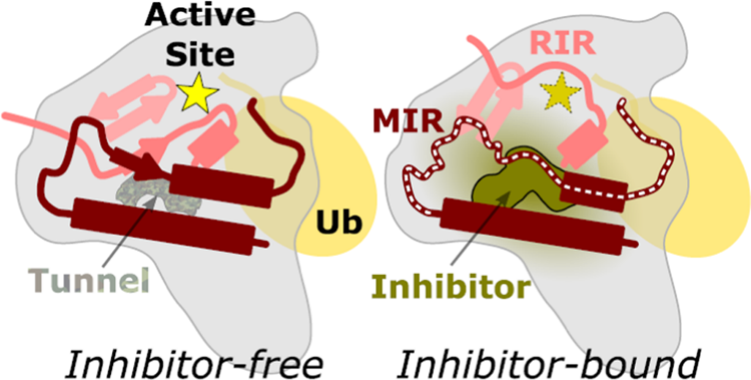

DNA damage triggers cell signaling cascades that mediate repair. This signaling is frequently dysregulated in cancers. The proteins that mediate this signaling are potential targets for therapeutic intervention. Ubiquitin-specific protease 1 (USP1) is one such target, with small-molecule inhibitors already in clinical trials. Here, we use biochemical assays and cryo-electron microscopy (cryo-EM) to study the clinical USP1 inhibitor, KSQ-4279 (RO7623066), and compare this to the well-established tool compound, ML323. We find that KSQ-4279 binds to the same cryptic site of USP1 as ML323 but disrupts the protein structure in subtly different ways. Inhibitor binding drives a substantial increase in thermal stability of USP1, which may be mediated through the inhibitors filling a hydrophobic tunnel-like pocket in USP1. Our results contribute to the understanding of the mechanism of action of USP1 inhibitors at the molecular level.

## Introduction

Ubiquitin-specific protease 1 (USP1) deubiquitinates substrates that are involved in DNA repair. It functions with a cofactor protein, USP1-associated factor 1 (UAF1), that stimulates enzymatic activity and assists substrate engagement.^1−3^ These substrates include the DNA clamps—proliferating cell nuclear antigen (PCNA) and FANCI-FANCD2, involved in translesion synthesis^4,5^ and the Fanconi Anemia pathway,^6−10^ respectively. Monoubiquitination of PCNA recruits polymerases that can bypass sites of DNA damage,^11^ while monoubiquitination of FANCI-FANCD2 appears to lock the complex onto chromatin.^7−10,12,13^ Deubiquitination by USP1 is presumed to reverse these processes to ensure genomic integrity during DNA repair.

USP1 is emerging as a potential target in the treatment of several cancers. It has long been postulated that synthetic lethality—simultaneous disruption of two or more nonessential genes/proteins resulting in cell death—may yield targeted cancer therapies.^14^ This is exemplified by poly(ADP-ribose) polymerase (PARP) inhibitors for the treatment of *BRCA1/2* mutant tumors.^15,16^ However, tumors may develop resistance through a number of mechanisms.^17^ The *USP1* gene is upregulated in several types of cancer, and this often correlates with poor prognosis,^18−22^ with some of these tumors also containing mutations in the *BRCA1* gene.^19^ Synthetic lethality between *USP1* and *BRCA1/2* has been demonstrated with clustered regularly interspaced short palindromic repeats (CRISPR)/CRISPR-associated protein 9 (CAS9) screens and USP1 inhibitors.^23,24^ The combination of PARP inhibitors and USP1 inhibitors in *BRCA1/2* mutant tumors is even more effective than PARP inhibitors or USP1 inhibitors alone in this genetic subpopulation^23,24^ and may provide a means for overcoming PARP inhibitor resistance. There are currently several USP1 inhibitors in phase 1 clinical trials – KSQ-4279/RO7623066 (KSQ Therapeutics/Hoffmann-La Roche), ISM3091/XL309 (In Silico Medicine/Exelixis), SIM0501 (Simcere Jiangsu Pharmaceutical Co), and HSK39775 (Xizang Haisco Pharmaceutical Co). Another USP1 inhibitor, TNG348 (Tango Therapeutics), was also in a phase I clinical trial but has recently been terminated due to liver toxicity. Mechanistically, PARP inhibitors block enzymatic activity and trap PARP1 on DNA;^25^ in fact, PARP1 inhibition is more cytotoxic than PARP1 removal.^25^ Curiously, there is evidence that USP1 can also become trapped on DNA when its enzymatic activity is impaired.^26^ However, disruption of PCNA homeostasis appears to be critical to the cytotoxicity of USP1 inhibition.^24^

**Figure 1:**
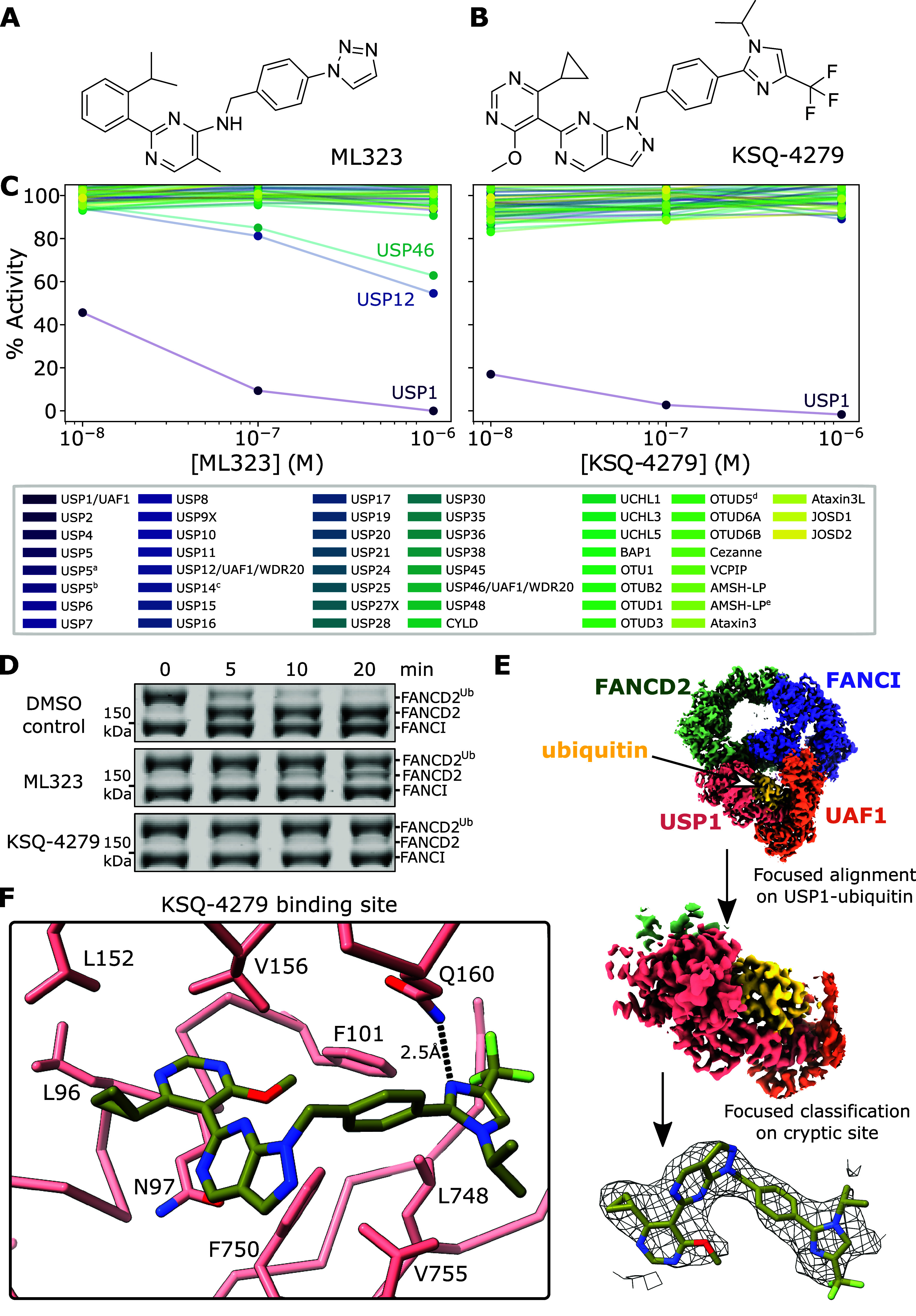
Biochemical and structural characterization of KSQ-4279. (A) Chemical structure of the tool compound, ML323. (B) Chemical structure of the clinical USP1 inhibitor, KSQ-4279. (C) Evaluation of the selectivity of ML323 and KSQ-4279 inhibitors across the DUBprofiler panel (Ubiquigent). ^a^Enzyme activated half-maximally with additional ubiquitin; ^b^enzyme activated maximally with additional ubiquitin; ^c^enzyme activated with proteasome-V5; ^d^pS177; and ^e^with additional zinc added. (D) Gel-based assay to demonstrate USP1 activity against FANCI-FANCD2^Ub^-dsDNA in the presence or absence of ML323 or KSQ-4279 (25 μM). USP1-UAF1 enzyme and FANCI-FANCD2^Ub^ substrate were used at 0.01 and 1 μM, respectively. Two technical replicates were performed. (E) Cryo-EM analysis of USP1^C90S^-UAF1-FANCI-FANCD2^Ub^-dsDNA with KSQ-4279. Density within 2.5 Å of KSQ-4279 is shown at 7.1σ (threshold of 0.085; sharpened map). (F) KSQ-4279 binding site (9FCI). Side chains of residues contacting KSQ-4279 and protein Cα atoms are shown; black dashed lines indicate hydrogen bonds.

The first selective USP1 inhibitor, ML323, was developed by the Zhuang and Maloney groups^27,28^ (Figure 1A). We recently determined the structure of this inhibitor in complex with USP1, UAF1, and the FANCI-FANCD2^Ub^ substrate using cryo-electron microscopy (cryo-EM).^29^ USP1 is inhibited by ML323 via interaction with a cryptic binding site, situated between the palm and thumb subdomains of the USP fold, that is almost completely obscured in the absence of inhibitor^29^ (PDB IDs: 7AY0, 7AY2,^3^7ZH3^29^). We sought to elucidate the binding mode of the clinical USP1 inhibitor, KSQ-4279 (Figure 1B), and compare this to that of ML323. We found that both ML323 and KSQ-4279 drive a substantial increase in the stability of the USP fold of USP1. Cryo-EM revealed a similar binding mode between the two compounds, with both compounds occupying a hydrophobic tunnel. KSQ-4279 drives subtle rearrangements in the cryptic site compared with ML323 and the compounds result in different levels of disorder in the adjacent regions. Our data are consistent with an induced fit binding to USP1 for both inhibitors.

## Results and Discussion

### Biochemical Comparison

We first compared ML323 and KSQ-4279 inhibition in ubiquitin-rhodamine assays using the DUB*profiler* assay (Ubiquigent). Screening against a panel of almost 50 deubiquitinase enzymes revealed that both ML323 and KSQ-4279 were selective against USP1 at 0.01 μM inhibitor (Figure 1C). KSQ-4279 retained exquisite selectivity for USP1 at inhibitor concentrations as high as 10,000 times the IC_50_ value (data not shown). In contrast, ML323 showed the inhibition of USP12 and USP46 at concentrations 100 times higher than the IC_50_ value for USP1. USP12 and USP46 are two close homologues of USP1 that also bind UAF1.^30−33^ Both ML323 and KSQ-4279 resulted in the near-complete inhibition of USP1-UAF1.

### Structural Characterization of KSQ-4279 Binding

**Figure 2:**
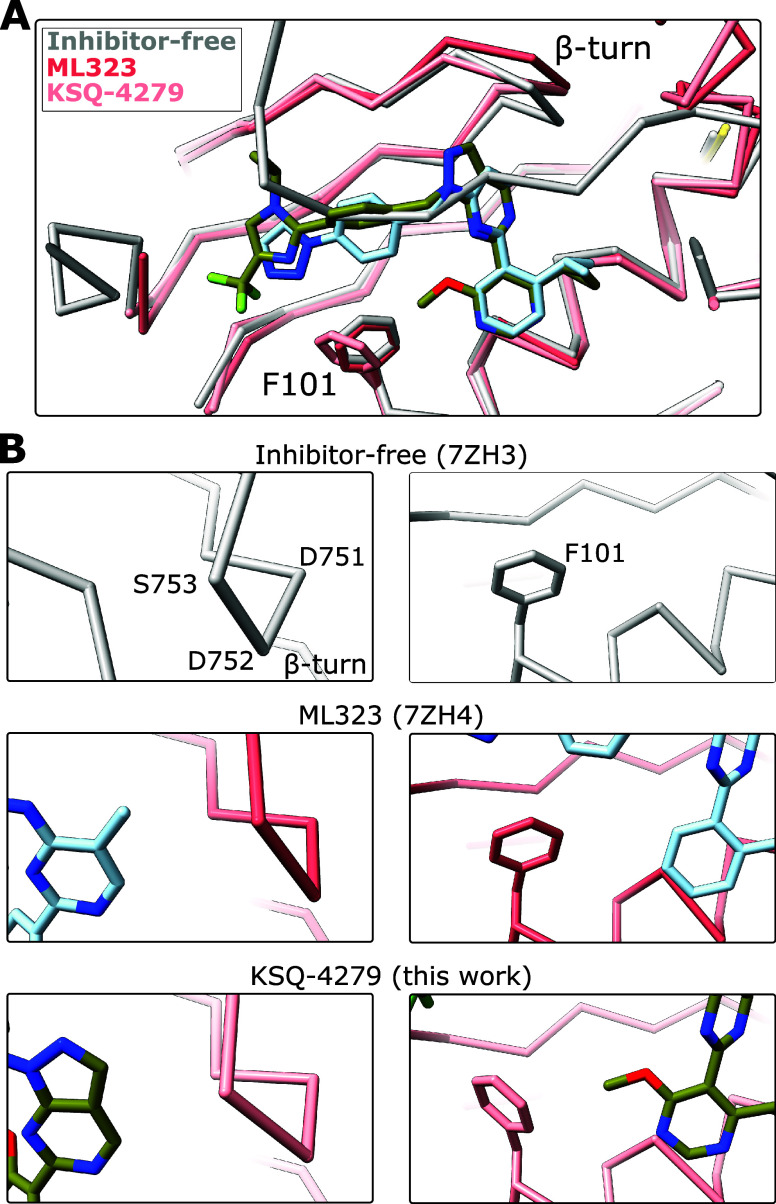
Differences in the effects of ML323 and KSQ-4279 on USP1 structure. (A) Superposition of the inhibitor binding sites for KSQ-4279 (olive; 9FCI) and ML323 (blue; 7ZH4). (B) KSQ-4279 perturbs the β-turn, on which catalytic aspartates D751 and D752 reside, to a lesser extent than ML323. KSQ-4279 perturbs F101, whereas ML323 does not. Protein backbone Cα atoms are shown.

In order to determine the structure of KSQ-4279 bound to USP1, we employed the FANCI-FANCD2^Ub^ substrate to trap a larger complex more amenable to cryo-electron microscopy, similar to our approach with ML323.^29^ Excess KSQ-4279 reduced the extent of deubiquitination of the FANCI-FANCD2^Ub^-dsDNA substrate in reconstitution, gel-based assays (Figures 1D and S1). After 20 min incubation with enzyme, almost no deubiquitination was observed in the presence of KSQ-4279, contrasting with results obtained in the presence of excess ML323 for which deubiquitinated Fanconi anemia group D2 protein (FANCD2) was apparent. This suggests that KSQ-4279 disrupts FANCI-FANCD2^Ub^-dsDNA deubiquitination to a greater extent than ML323. Cryo-EM analysis of the C90S active site mutant of USP1 reconstituted with its cofactor UAF1 and FANCI-FANCD2^Ub^-dsDNA substrate, in the presence of KSQ-4279, allowed reconstruction of USP1-ubiquitin with this inhibitor at a resolution of ∼3.2 Å (Figures 1E, S2, and Table S1). The data set contained both inhibitor-bound and inhibitor-free particles and three-dimensional (3D) classification was used to identify a subset consistent with inhibitor-bound USP1, similarly to ML323^29^ (Figure S2). KSQ-4279 binds the same cryptic pocket as ML323, between the palm and thumb subdomains, displacing several residues of the hydrophobic core (Figures 1F and 2A). We refer to these residues, 76–88 of USP1, as the replaced by inhibitor region (RIR). The USP1-bound conformation of KSQ-4279 is similar to that of ML323 (Figure 2A). The 4-cyclopropyl-6-methoxypyrimidin-5-yl group of KSQ-4279 occupies the same position as the 2-propan-2-ylphenyl group in ML323. The 1-propan-2-yl-4-(trifluoromethyl)imidazol-2-yl moiety is slightly shifted compared to the triazol-1-yl group of ML323 (RMSD of ring atoms ∼0.9 Å). The phenyl group of KSQ-4279 is rotated with respect to the ML323 structure; however, given the limits of the resolution, this is within the uncertainty of the modeling. N160 of USP1 is within the hydrogen bonding distance of KSQ-4279 (Figure 1F), similar to the ML323 structure.^29^ The β-turn on which the catalytic aspartates, D751 and D752,^34^ reside is pushed by the pyrazolo[3,4-*d*]pyrimidine group of KSQ-4279, slightly displacing these residues with respect to the inhibitor-free USP1 (Figure 2). In ML323, the methyl substituent of the pyrimidine also pushes this β-turn but to a greater extent, also displacing S753. F101 is pushed out slightly to accommodate the methoxyl substituent of KSQ-4279, which was not observed for ML323-bound USP1 (Figure 2). Overall, there are subtle changes in the cryptic binding site between KSQ-4279 and ML323.

**Figure 3:**
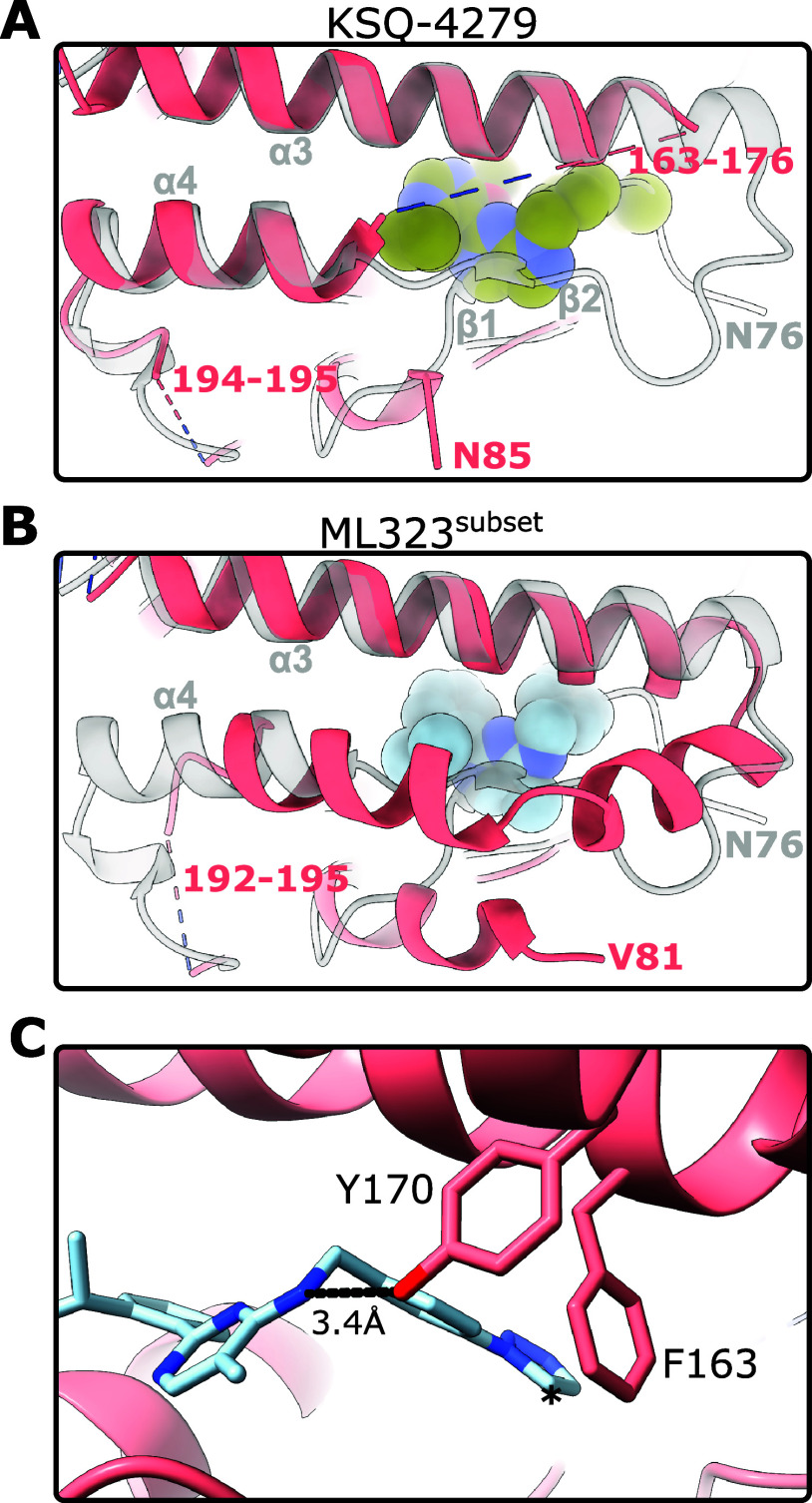
Comparison of the USP1 MIR between ML323 and KSQ-4279. (A) KSQ-4279 disrupts β-strands (β1 and β2) of the inhibitor-free state leaving residues 163–176 disordered (9FCI). The KSQ-4279-bound protein structure is shown in pink and the inhibitor-free structure (7ZH3^29^) in transparent gray. KSQ-4279 is shown as space-filling spheres based on van der Waals radii. The first ordered residue of the KSQ-4279-bound structure, N85, and the inhibitor-free structure, N76, are highlighted. (B) A subset of ML323-bound particles has reordered residues 167–191 resulting in the formation of a new helix and slipping of helix α4, in addition to bending of helix α3 (9FCJ). The ML323-bound protein structure is shown in pink, and the inhibitor-free structure (7ZH3^29^) is shown in transparent gray. ML323 is shown as space-filling spheres based on van der Waals radii. (C) Additional protein-inhibitor interactions of the ML323^subset^ structure (9FCJ). Dashed lines indicate potential hydrogen bond. The asterisk indicates where the isopropyl group of KSQ-4279 would clash with F163. ML323 is shown as sticks.

The region with high structural heterogeneity in the ML323-bound structure,^29^ spanning residues 168–195, also exhibits reduced order in the KSQ-4279-bound structure (Figure 3A). However, unlike the ML323 structure, helix α4 in this region remains in the same position as in the inhibitor-free structure, and the region between α3 and α4 is poorly resolved and was left unmodelled. We refer to residues 168–195 as the mobilized by inhibitor region (MIR). Comparing the KSQ-4279 and ML323 maps, the density in the ML323 map resembles a combination of that observed in the KSQ-4279 structure and a conformation where α4 has slipped (Figure S3). We therefore sought to identify subsets of particles in our previous ML323 data set (EMPIAR-11299^29^) in which the MIR was sufficiently resolved. We managed to isolate a subset of particles from which we could build an atomic model (ML323^subset^; Figure S4). In the ML323^subset^ structure, helix α4 slips closer to the inhibitor binding site by approximately 1.5 turns and the region between helix α3 and α4 (including β2) reorders to form another helix (Figure 3B). In both inhibitor-bound structures, residues near G194 are structurally heterogeneous. A similar region in USP7 also exhibits plasticity and has been referred to as the switching loop.^35^ This short stretch (residues 192–195 in USP1) may be innately flexible in the USP fold.

In the ML323^subset^ MIR, Y170 of the newly formed helix likely hydrogen-bonds with the secondary amine joining the pyrimidine and benzyl groups, while F163 is brought close to the triazole group (Figure 3C). However, KSQ-4279 lacks a secondary amine at the equivalent position and has substituents that would clash with F163. As such, the conformation of the ML323^subset^ USP1 structure is incompatible with the KSQ-4279 molecule. This may explain the lack of order in this region when KSQ-4279 is bound. Aside from the major conformational changes required to establish the cryptic site, it appears that KSQ-4279 binding maintains the USP1 fold in a slightly more native conformation than the binding of ML323.

**Figure 4:**
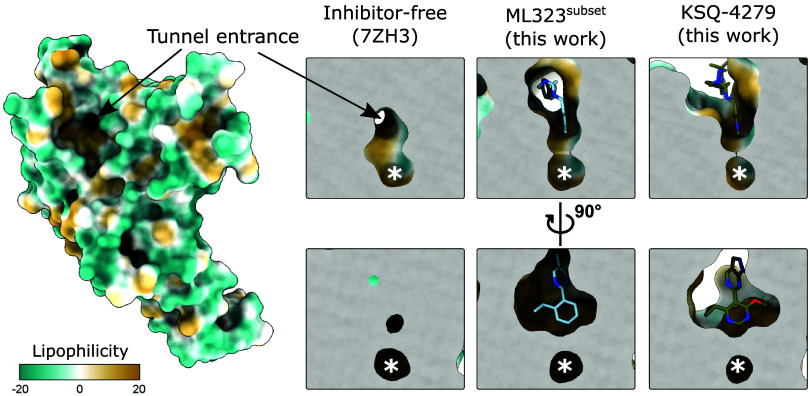
A hydrophobic tunnel is occupied by USP1 inhibitors. Solvent-excluded surface of inhibitor-free USP1 (7ZH3,^29^ USP1 chain) (left). KSQ-4279 and ML323 binding leaves only a small cavity remaining (asterisk). Note that the MIR was left unmodeled in the KSQ-4279 structure and would sit on top of KSQ-4279. Solvent-excluded surfaces are colored by lipophilicity, and inhibitors are shown as sticks. Each inhibitor-bound structure was superposed onto the inhibitor-free structure (7ZH3^29^).

In the absence of inhibitor, there is a hydrophobic tunnel-like pocket near where the inhibitors bind (Figure 4). Binding of ML323 or KSQ-4279 rearranges and almost completely fills this tunnel to form the cryptic site. The tunnel is primarily plugged by the 2-propan-2-ylphenyl group in ML323 and the 4-cyclopropyl-6-methoxypyrimidin-5-yl group in KSQ-4279. The presence of this tunnel may facilitate preliminary binding to USP1 and direct conformational changes required to generate the cryptic site.

Both inhibitors leave a small hydrophobic cavity (Figure 4, asterisk), raising the possibility for the expansion of the inhibitors into this cavity. This could potentially be achieved via a substituent with linear geometry at the 2-position of the pyrimidine of KSQ-4279, such as a propyne group. Additionally, in USP12 and USP46, this cavity is blocked off (Figure S5). As such, selectivity may be improved in inhibitors that fill the cavity.

**Figure 5:**
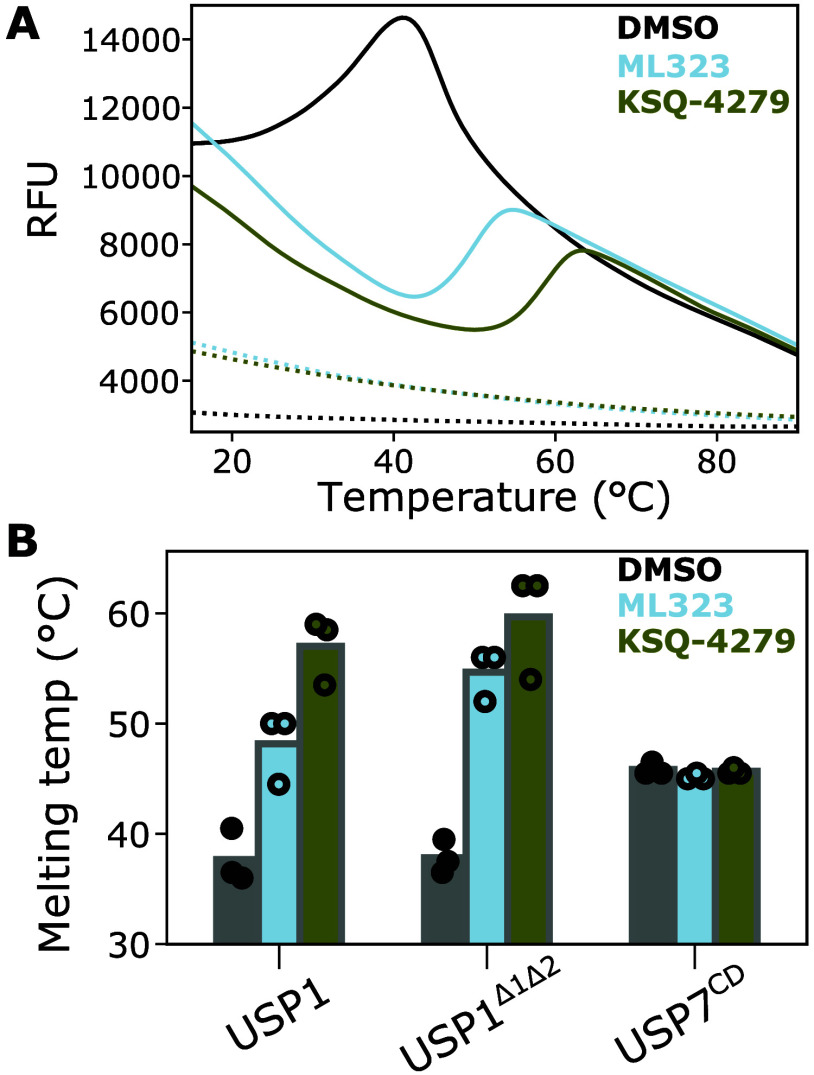
Inhibitor binding stabilizes the USP fold. (A) Thermal shift assays of USP1 (3.8 μM) in the presence or absence of ML323 or KSQ-4279 (50 μM) (solid lines). ML323, KSQ-4279, and DMSO without USP1 are shown as dashed lines. (B) Quantification of thermal shift assays using the inflection point to estimate the melting temperature. USP7 catalytic domain (USP7^CD^) was included as a negative control. At least two technical replicates for each USP were performed and are shown as circles.

Using thermal shift assays, we compared the effect of inhibitors on the isolated USP1 protein (Figure 5). Strikingly, an excess of ML323 and KSQ-4279 increased the melting temperature by 11 and 19 °C, respectively. Both full-length USP1, and USP1 with the disordered inserts 1 and 2 deleted (USP1^Δ1Δ2^; construct details in the Experimental Section) were shifted to similar extents (Figure 5B). This is consistent with stabilization of the USP fold itself rather than perturbing interaction with inserts 1 and 2. In contrast, the USP7 catalytic domain, used here as a control, did not show any change in the melting temperature in the presence of either inhibitor. Overall, this is consistent with the inhibitors filling the hydrophobic tunnel to create a more stable hydrophobic core of USP1.

### Effect of Inhibitors on Catalysis

**Figure 6:**
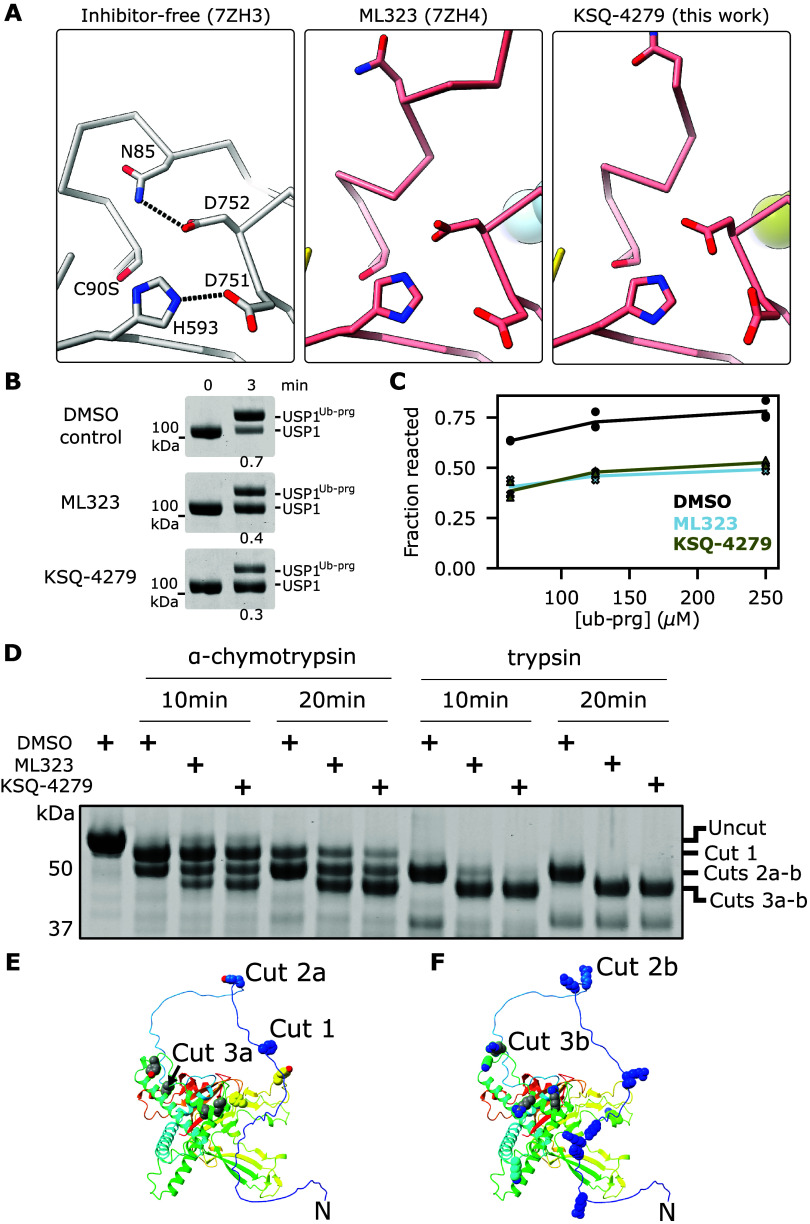
Disruption of USP1 active site. (A) ML323 or KSQ-4279 binding disrupts hydrogen bonding of the catalytic aspartates and displaces the oxyanion stabilizing residue, N85. Hydrogen bonds of the inhibitor-free structure are shown as dashed lines. (B) Gel-based assay for reactions of USP1 (2 μM) in the presence or absence of ML323 or KSQ-4279 (25 μM) with Ub-Prg (6 μM) at room temperature. Fraction of signal corresponding to the reacted band is given below each reaction. Two technical replicates were performed. (C) Gel-based assay of reactions of 2 μM USP1 alone or with 25 μM inhibitor with excess Ub-Prg on ice using a single 3 min time-point. Fraction reacted was quantified using densitometric analysis of the bands. At least two technical replicates were performed. (D) Limited proteolysis reactions of USP1^Δ1Δ2^ (10 μM) with different proteases in the presence or absence of ML323 or KSQ-4279 (25 μM). (E) AlphaFold model of USP1^Δ1Δ2^ with residues of highly probable α-chymotrypsin cleavage sites and high solvent accessibility shown as spheres. Residues with high cleavage probability that are only accessible in inhibitor-bound structures are colored gray. The N-terminus and probable sites for cleavage products generated by cuts 1–3 are indicated. (F) AlphaFold model of USP1^Δ1Δ2^ with residues of highly probably trypsin cleavage sites and high solvent accessibility shown as spheres. Residues with high cleavage probability that are only accessible in inhibitor-bound structures are colored gray. The N-terminus and probable sites for cleavage products generated by cuts 2 and 3 are indicated. Protein cartoons are colored by Jones’ rainbow.

The catalytic site of the KSQ-4279-bound structure was perturbed as previously observed for ML323^29^ (Figure 6A). The β-turn on which D751 and D752 reside is displaced; however, the density for the carboxyl groups of D751 and D752 was not sufficient to unambiguously assign these atoms in the KSQ-4279-bound structure. Regardless, the hydrogen bonding between H593 and D751 is disrupted. Recently, both D751 and D752 have been demonstrated to be important for USP1 catalysis.^34^ Therefore, inhibition by KSQ-4279 and ML323 may not be as simple as our previous hypothesis of stabilizing a flipped H593 conformation that cannot efficiently deprotonate C90, thereby reducing the catalytic cysteine’s ability to exert a nucleophilic attack at the isopeptide bond.^29^ Indeed, N85 is involved in the stabilization of the oxyanion intermediate during catalysis for other USPs^37,38^ and it forms a hydrogen bond with D752 in USP1 in the absence of any inhibitor (Figure 6A). Binding of either inhibitor not only disrupts this bonding but also displaces N85 altogether. As such, inhibitor binding may not only impede the coordination of H593 but also destabilize the oxyanion intermediate.

To examine the effect of inhibitor on the early stages of catalysis, we used ubiquitin-propargyl amide (Ub-Prg) reactions. The catalytic cysteine of deubiquitinases can nucleophilically attack the Ub-Prg probe to yield a stable covalent adduct.^39,40^ Reaction of Ub-Prg with inhibitor-free USP1 at room temperature was ∼70% complete within 3 min (Figure 6B). In the presence of an excess of either inhibitor, the reaction between Ub-Prg and USP1 was reduced, with ∼30 to 40% USP1 reacted at 3 min. The reduced reaction with Ub-Prg in the presence of inhibitor at 3 min may be due to reduced Ub-Prg binding rate or reduced reaction rate. To increase the Ub-Prg binding rate such that this step is not rate-limiting, we used >50 μM Ub-Prg (>30-fold molar excess over USP1). We performed reactions on ice to enable single time-point measurements at 3 min. Both ML323 and KSQ-4279 reduced the reaction with Ub-Prg to a similar extent under these conditions (Figure 6C). This result is consistent with the binding of ML323 or KSQ-4279 perturbing nucleophilic attack of the catalytic cysteine; however, the oxyanion hole may also be important in this reaction.^41^

The oxyanion stabilizing residue, N85, is located on the RIR of USP1. We hypothesize that its displacement upon the binding of ML323 or KSQ-4279 would make the RIR susceptible to more generic proteases. Therefore, we explored limited proteolysis assays to characterize inhibitor binding (Figures 6D and S6). We treated USP1^Δ1Δ2^ with α-chymotrypsin and found that the protein was truncated to two slightly smaller products in the absence of an inhibitor. Treatment with trypsin resulted in one slight truncation in the absence of inhibitor. In the presence of ML323 or KSQ-4279 an additional, faster migrating band was also present for both proteases, indicating further truncation. Considering the cleavage site preferences for α-chymotrypsin and trypsin and the accessibility of the sites based on an AlphaFold model of USP1^Δ1Δ2^ (Figures 6E,F and S7), we interpret the second truncated species to be cleaved at Y52 (Cut 2a) or R53 (Cut 2b) for α-chymotrypsin and trypsin, respectively, and the third species to be cleaved at F80 (Cut 3b) or R74 (Cut 3a) for α-chymotrypsin and trypsin, respectively. Cuts 3a-b only occur in the presence of ML323 or KSQ-4279 which is consistent with inhibitor binding displacing the RIR containing N85 and making R74 and F80 more accessible to the proteases. Lack of cleavage at the R74, F80 sites in the absence of inhibitor, suggests this region is not significantly exposed in the absence of inhibitor, consistent with an induced fit mode of binding within the hydrophobic tunnel.

### Comparison with Other USPs and Inhibitors

Given the improved selectivity of KSQ-4279 over ML323 for USP12 and USP46 (Figure 1C), we sought to rationalize this from the structures. We aligned USP12^33^ and USP46^31^ to inhibitor-free USP1 and superposed the KSQ-4279 and ML323-bound structures onto these (Figure S8). We looked for protein-inhibitor clashes in the regions that remain fully ordered in USP1 upon inhibitor binding, i.e., excluding the MIR and RIR. ML323 clashes with N97 and the β-turn on which the catalytic aspartates reside^34^ in all three USPs. KSQ-4279 clashes with N97 in all three USPs and the same β-turn that ML323 clashes with in USP12. A further clash for KSQ-4279 is observed in all superpositions between the methoxyl group and F101 (F59 and F55 in USP12 and USP46, respectively). We therefore propose that the rearrangement of F101 in USP1 (Figure 2B) cannot be as easily accommodated in USP12 and USP46, as a mechanism for the increased selectivity of KSQ-4279 over ML323. However, this mechanism assumes that the region homologous to USP1’s MIR also becomes flexible in USP12 and USP46. We cannot rule out that interactions between the 1-propan-2-yl-4-(trifluoromethyl)imidazol-2-yl group and the regions homologous to the MIR, which are much shorter in USP12 and USP46, mediate the difference in the selectivity between ML323 and KSQ-4279.

Based on this proposal, dual substitutions at positions 4 and 6 of the pyrimidine moiety of KSQ-4279 would be important to expand into the cryptic site. Both ML323 and another related USP1 inhibitor, I-138, contain a single substitution in the equivalent region (2-propan-2-ylphenyl group). I-138 has been reported to inhibit USP12 and USP46 by ∼50% at 10 μM,^24^ while KSQ-4279 shows <50% inhibition against these two USPs even at 100 μM (Figure S9). This selectivity difference is consistent with dual substituents in this region mediating greater selectivity. Furthermore, in a recent SAR study of USP1 inhibitors, dually substituted rings in the equivalent region tended to have improved IC_50_ values.^42^ Of note, TNG348 also contains a dually substituted pyrimidine, equivalent to KSQ-4279. At the opposite extremity of the inhibitors, various groups are well tolerated in terms of IC_50_ values;^28^ however, substituents tend to improve inhibition.^42^ The central portion of ML323 contains a methylpyrimidin-4-amine, while in other USP1 inhibitors, this is cyclized in various different ways.^24,28,42^ The choice of cyclization strategy does not seem to dramatically impact activity. However, different cyclization strategies may displace the β-turn on which the catalytic aspartates reside to different extents. In particular, TNG348 has a trifluoroethyl substituent that is expected to displace the β-turn even further. The apparent plasticity in the β-turn suggests the potential for adding groups that could hydrogen bond with the β-turn backbone, particularly the carbonyl of D752. Finally, the interactions between the MIR and ML323 that are incompatible with KSQ-4279 are also incompatible with the TNG348 and I-138 compounds and those of Li et al.,^42^ suggesting that reordering of the MIR is not essential for efficient inhibition.

Next, we sought to identify how USP1, USP12, and USP46 may be selected over other USPs. We hypothesized that the hydrophobic tunnel of USP1, which is also present to some extent in USP12 and USP46 (Figure S5), may be absent in other USPs. We aligned 48 AlphaFold predictions and the KSQ-4279 structure by the USP fold and looked for pockets near the pyrimidine of KSQ-4279 (Figure S10). Consistent with the experimental structures, AlphaFold predictions of USP12 and USP46 have a small cavity in this region. However, so too does USP11 which was not inhibited by either compound at the concentrations tested (Figure 1C), therefore the presence of a hydrophobic tunnel in this region does not appear to exclusively mediate the selectivity.

Finally, we assessed the nonselective USP1 inhibitor, SJB2–043,^43^ using similar assays (Figure S11). This compound is structurally distinct from the inhibitors discussed above (Figure S11A). In deubiquitination assays with K48-linked diubiquitin substrate and USP1^Δ1Δ2^ enzyme, inhibition by SJB2–043 is much more modest than ML323 or KSQ-4279 (Figure S11B). Consistent with this result, the reduction in reaction with excess Ub-Prg is also less than that with ML323 or KSQ-4279 (Figure S11C). Furthermore, thermal melting of USP1 in the presence of SJB2–043 does not show the same increase in stability as ML323 or KSQ-4279 (Figure S11D), and the limited proteolysis profile does not show Cut 3 (Figure S11E). These data suggest SJB2–043 acts through a fundamentally different mechanism, consistent with the lack of selectivity of related compounds for USP1.^43,44^

### Limitations

The extensive protein rearrangements to establish the cryptic site suggest plasticity in the binding site. The differences between the ML323 and KSQ-4279-bound structures are consistent with this, particularly at F101 and the β-turn (Figure 2B) and the MIR (Figures 3 and S3). These variations between the inhibitor-bound structure likely underlie the difference in melting temperatures observed when USP1 is bound to ML323 versus KSQ-4279 (Figure 5). As such, additional structural data will likely be necessary to develop a reliable pharmacophore model.^36^

The structures of inhibitor-bound USP1 were determined in the context of the C90S mutation of USP1, and USP1 is bound to the substrate, which may perturb the enzyme structure compared to substrate-free wild-type enzyme. However, we were unable to obtain reliable structures of USP1-UAF1 alone with the inhibitor. Despite the presence of a substrate, the extensive inhibitor interactions at the cryptic binding site are almost certainly similar in the absence of a substrate. As our data contained a mixture of inhibitor-bound and inhibitor-free states, we used classification algorithms to sort the particles. However, cryo-EM data is inherently noisy and these algorithms are imperfect on such data; therefore, there is the potential for misclassification to perturb the resulting reconstructions. We explored many classification parameters to mitigate this. In the KSQ-4279 structure, the density for N97, adjacent to the cryptic site, is poor. This may be a manifestation of misclassified particles, additional substates, or possible radiation damage. Finally, the reconstruction resolutions of the ML323 and KSQ-4279 structures vary, which complicates the comparison of the maps. In particular, we are unable to reliably model waters in the KSQ-4279 structure. For comparison of the MIR, the maps were low-pass filtered to 5 Å to mitigate the effect of the difference in resolutions.

## Conclusions

Overall, our data suggest that the clinical USP1 inhibitor, KSQ-4279, and well-established tool compound ML323 exert similar inhibitory effects on USP1. Both inhibitors bind to a cryptic binding site in a similar mode. However, the surrounding residues of the protein are disrupted in different ways. KSQ-4279 is clearly more selective than ML323 with respect to its effect on other USP enzymes. KSQ-4279 may exhibit greater selectivity for USP1 over USP12 and USP46 compared to ML323 due to the methoxyl substituent of KSQ-4279 further disturbing the binding site; however, this requires further investigation. The occupancy of a hydrophobic tunnel by the inhibitors appears to drive a substantial increase in the thermal stability of USP1. This work helps to define the mechanism of action of USP1 inhibitor KSQ-4279, currently in clinical trials.

## Experimental Section

### Protein Expression and Purification

Proteins, all human homologues, were expressed in either insect cells or bacteria as described previously.^3,45^ Protein purification buffers and columns used are listed in Table S2. Briefly, His_6_-TEV-USP1^G670A,G671A^, His_6_-TEV-USP1^G670A,G671A,C90S^, His_6_-TEV-USP1^Δ229–408,Δ608–737^ (USP1^Δ1Δ2^), His_6_-3C-UAF1, His_6_-3C-FANCD2, His_6_-TEV-V5-FANCI, and His_6_-TEV-V5-FANCI^S556A,S559A,S565A^ were expressed separately in *Sf*21 insect cells. Cells were lysed by sonication, clarified, and purified by Ni-NTA affinity and then anion exchange chromatography. At this stage, protein aliquots were occasionally flash-frozen in liquid nitrogen and stored at −80 °C. For His_6_-TEV-USP1^G670A,G671A^, His_6_-TEV-USP1^G670A,G671A,C90S^, and His_6_-TEV-USP1^Δ229–408,Δ608–737^ (USP1^Δ1Δ2^), tobacco etch virus (TEV) protease treatment was performed overnight at ∼1:10 protease to target protein with gentle agitation, resulting in cleaved protein with an N-terminal glycine extension. Subtractive Ni-NTA affinity chromatography was subsequently performed. All proteins were concentrated to 5–10 mg/mL and separated by gel filtration. Purified protein was concentrated to 5–15 mg/mL, flash-frozen, and stored at −80 °C in 10–20 μL single-use aliquots. All steps were performed on ice or at 4 °C and completed within 24–36 h of lysis. FANCD2 was ubiquitinated and purified using an engineered Ube2T and SpyCatcher-SpyTag setup described in detail elsewhere.^46^ For FANCD2, the His_6_-3C tag was removed by 3C protease treatment during the preparation of the monoubiquitinated version.

The USP domain from USP7 (residues 208–560) was expressed as a His_6_-smt3 fusion in BL21 *Escherichia coli* cells. Cells were grown to an OD_600_ of ∼0.6 and expression induced with 0.1 mM Isopropyl β-d-1-thiogalactopyranoside (IPTG) at 16 °C for ∼18 h. Cells were harvested and lysed by sonication. Lysate was clarified and bound to Ni-NTA resin. His_6_-ULP1 protease was added to the resin and incubated overnight at 4 °C to cleave the His_6_-smt3 tag. Flow-through was collected and purified by anion exchange chromatography, followed by gel filtration. Purified protein was concentrated to ∼10 mg/mL, flash-frozen, and stored at −80 °C in 10–20 μL single-use aliquots.

Ubiquitin-propargylamine (Ub-Prg) was prepared with an N-terminal twin-strep tag. Ubiquitin^1–75^-Intein-Chitin binding domain (CBD) plasmid was gifted from Dr Yogesh Kulathu (University of Dundee) and a twin-Strep and a 3C cleavage site was cloned N-terminally. Twin-strep-3C-ubiquitin^1–75^-Intein-CBD was produced in BL21 *E. coli* cells. Cells were grown to an OD_600_ of 0.4–0.5 and expression induced with 0.3 mM IPTG at 16 °C for ∼24 h. Cells from 6 L expression culture were harvested, lysed by sonication, clarified, and bound to chitin beads. Beads were washed with ∼40× bead volume of Wash Buffer 1 followed by 10× bead volume of Wash Buffer 2. Intein autocleavage was performed in three elution steps, each with >2.5× bead volume of Elution Buffer and >24 h incubation. Eluant was concentrated to ∼20 mg/mL and pH-adjusted to 8.0 with 0.1 M NaOH (or dialyzed into 50 mM HEPES). The propargylamine (prg) warhead was then conjugated by incubating with 0.25 M propargylamine 4–6 h at 16 °C in the dark and mild agitation at 30 min intervals. Ub-Prg was concentrated and separated by SEC using an SD75 16/600 column in 1× PBS before concentrating to ∼10 mg/mL and storage at −80 °C.

Protein concentrations were determined using the predicted extinction coefficients at 280 nm^47^ and absorbance via a NanoDrop. The ratio of 260/280 nm was ≤0.65 for all protein batches used in subsequent experiments.

### Inhibitors

Compounds were purchased from MedChemExpress and used without further purification. Solutions of up to 100 mM were prepared by dissolving the compounds in DMSO. All compounds were >95% pure by HPLC.

### Ubiquitin-Rhodamine Assays

To determine the potency of both compounds on USP1, the ability of USP1-UAF1 (0.008 nM) to cleave the ubiquitin-rhodamine substrate (100 nM) was determined in the presence or absence of a half-log, eight-point dilution series of each compound. IC_50_ values were determined from the dose–response curve of each compound by fitting the Hill equation.

The selectivity of KSQ-4279 and ML323 was then evaluated in the DUB*profiler* assay (Ubiquigent) against 48 individual deubiquitinase enzymes, at concentrations of 0.01, 0.1, 1, 10, and 100 μM of each compound, the latter being equivalent to at least 10,000-fold greater concentrations than the IC_50_ against the primary target.

Values in the presence of compound were compared with those of DMSO controls to give the percentage of remaining activity. Neither compound showed any significant autofluorescent properties in the ubiquitin-rhodamine assay up to 100 μM.

### Cryo-EM Sample Preparation

The USP1^C90S^-UAF1-FANCI-FANCD2^Ub^ complex was prepared by mixing the four individually purified subunits at 5:5:1:1 (USP1^G670A,G671A,C90S^:His_6_-3C-UAF1:His_6_-TEV-V5-FANCI^S556A,S559A,S565A^:FANCD2^Ub^) as described previously.^29^ The complex was exchanged into EM buffer (20 mM Tris pH 8.0, 150 mM NaCl, and 2 mM DTT) using a Bio-Spin P-30 column (Bio-Rad). The concentration of the complex was estimated from absorbance at 280 nm (assuming no loss of any of the protein components). 1.2 equiv of dsDNA per FANCI-FANCD2^Ub^ was then added (61 base pairs; TGATCAGAGGTCATTTGAATTCATGGCTTCGAGCTTCATGTAGAGTCGACGGTGCTGGGAT; IDT). Finally, KSQ-4279 was added at 2 equiv of USP1-UAF1. The sample was incubated at room temperature for 5 min immediately prior to preparing grids. UltrAuFoil R1.2/1.3 300 mesh grids were glow-discharged at 35 mA for 60 s. A 3.0 μL aliquot of 9.6 μM USP1-UAF1, 1.9 μM FANCI-FANCD2^Ub^, 2.3 μM dsDNA, 18.8 μM ML323 was applied. The grids were blotted for 3.0 s and vitrified in liquid ethane using a Vitrobot (Thermo Fisher) operating at ∼95% humidity at 15 °C.

### Cryo-EM Sample Data Collection and Processing

Grid screening and data collection were performed on a Titan Krios (Thermo Fisher) located at eBIC (Diamond Light Source) equipped with a K3 detector and BioQuantum Energy Filter (Gatan). A total of 6581 movies were collected using Beam-Image shift. Movies were collected in Counted Super Resolution with 2× binning and CDS at a pixel size of 0.83 Å using EPU (Thermo Fisher). An energy filter slit width of 20 eV was used. Movies were collected with a total dose of ∼62 e^–^/Å^2^ over 50 frames at a rate of ∼6 e^–^/px/s.

Subsequent processing was performed in cryoSPARC v3.3 and v4.4^48^ (Figure S2). Patch motion correction, patch CTF estimation, and manual curation were performed resulting in 5675 dose-weighted, motion-corrected micrographs. Both template picking and topaz^49^ were used to identify potential particles which were extracted at 384 × 384 pixels Fourier cropped to 128 × 128. Multiple two-dimensional (2D) classifications and heterogeneous refinement were used in parallel for initial cleaning with selected classes pool and duplicates removed. Further particle cleaning was performed using heterogeneous refinement with one good starting model and 5 “junk” starting models distinct from the protein complex of interest, all low-pass filtered to 20 Å. A final round of heterogeneous refinement was performed using the same starting model low-pass filtered at 12 Å, and two copies at 30 Å. Five full passes through the particle sets, after O-EM iterations, were used in heterogeneous refinements. Particles corresponding to the highest resolution class were subjected to another round of duplicates removal and were then re-extracted with a box size of 384 × 384 pixels without Fourier cropping. Nonuniform refinement^50^ yielded a structure with a global resolution of 3.6 Å from 135,680 particles. These were subjected to reference-based motion correction and further nonuniform refinement yielding a structure with a global resolution of 3.5 Å from 135,646 particles. Local refinement was performed with a mask covering USP1 and ubiquitin using a Gaussian prior of 3° over rotation and 2 Å over shifts with marginalization and nonuniform refinement. 3D classification was performed with a smaller mask covering the cryptic binding site and adjacent regions. Classes with density consistent with inhibitor binding were pooled and subjected to another round of local refinement with a mask covering USP1 and ubiquitin.

For reprocessing of the previously published data set with ML323 (EMPIAR-11299),^29^ reference-based motion correction was performed on particles from the consensus reconstruction prior to local motion correction (Figure S4). Local refinement was performed with a mask covering USP1 and ubiquitin using a Gaussian prior of 3° over rotation and 2 Å over shifts with marginalization and nonuniform refinement. 3D classification was performed with a mask covering the inhibitor binding site and 10 classes using “simple” initialization. A class with interpretable density for residues 168–191 (ML323^subset^) was passed through local refinement again, with the mask covering USP1 and ubiquitin.

### Model Building and Refinement

KSQ-4279 and ML323^subset^ structures were built using USP1 and ubiquitin using the previous structure of the ML323-bound structure (PDB ID: 7ZH4) as an initial model. Manual model editing was performed using COOT^51^ and ISOLDE.^52^ KSQ-4279 restraints were calculated using the GRADE web server (http://grade.globalphasing.org/). Automated refinement against the globally sharpened maps, with B-factors estimated from the Guinier plot, was performed using phenix real-space refinement.^53^ A refinement resolution of 3.8 and 3.2 Å was used for the KSQ-4279 and ML323^subset^ structures, respectively. Bond and angle restraints for the USP1 Zinc finger, as well as Ramachandran restraints, were incorporated during automated refinement. Cryo-EM data and model statistics are listed in Table S1. The FSC between the models and maps were computed using phenix^54^ (Figure S2). Structures and maps were aligned and analyzed, and figures were produced using ChimeraX.^55^

### Deubiquitination Assays

Deubiquitination reactions were performed as described previously.^29^ A 2× substrate mix and a 2× enzyme mix were prepared separately and mixed in a 1:1 ratio to initiate the reaction. Both mixes were set up on ice and then incubated at room temperature for at least 20 min prior to reaction initiation and during the reaction.

The 2× substrate mix was prepared by diluting stocks of FANCD2^Ub^ (>29 μM), His_6_-V5-TEV-FANCI (>50 μM), and dsDNA (100 μM; 61 base pairs) with DUB buffer (20 mM Tris pH 8.0, 75 mM NaCl, 5% glycerol, 1 mM DTT). The resulting 2× mix was composed of 2 μM FANCD2^Ub^, 2 μM Fanconi anemia group I protein (FANCI), and 8 μM dsDNA. The 2× enzyme mixes were prepared by diluting concentrated stocks (≥30 μM) of USP1, His_6_-3C-UAF1, and ML323 or KSQ-4279 or DMSO control with DUB buffer. The resulting 2× enzyme mixes were composed of 200 nM USP1^G670A,G671A^, 200 nM UAF1, 1% DMSO, and 50 μM inhibitor, where included. Assays with K48-linked diubiquitin were performed similarly, with 10 μM K48-linked diubiquitin and 100 nM USP1^Δ229–408,Δ608–737^ (USP1^Δ1Δ2^) in the 2× substrate mix and 2× enzyme mix, respectively. Aliquots of 4 μL of reaction were terminated at the indicated time points by the addition of 20 μL of 1.2× NuPAGE LDS buffer (Thermo Fisher) supplemented with 120 mM DTT. SDS-PAGE was performed using Novex 4–12% Tris-glycine gels or Novex 12% Bis-Tris gels (Thermo Fisher) and subsequent staining of the gels with Instant-Blue Coomassie stain (Expedeon). Gels were imaged on an Odyssey CLx (LI-COR) using the 700 or 800 nm channel. Densitometric analysis was performed using Image Studio (LI-COR), quantifying each band, and assuming similar staining properties between the ubiquitinated and deubiquitinated species.

### Thermal Shift Assays

Concentrated stocks of USP1^G670A,G671A^, USP1^Δ229–408,Δ608–737^ (USP1^Δ1Δ2^), or USP7^208–560^ (USP7^CD^) (>100 μM) were diluted to 4 μM with TSA buffer (50 mM Tris pH 8.0, 200 mM NaCl, 5% glycerol, 1 mM DTT). This was added to SYPRO Orange and inhibitor or DMSO control to yield a final concentration of 3.8 μM protein, 2–5× SYPRO Orange, 50 μM inhibitor, 1% DMSO in Thermo-Fast 96 skirted plates or hard-shell thin wall PCR plates. Thermal shift assays were performed using a CFX96 Touch Real-Time PCR Detection System (Bio-Rad) with 50 μL of sample heated from 15 to 90 °C at 3 °C/min and with fluorescence measured at 0.5 °C intervals. Data were analyzed using CFX Maestro (Bio-Rad) and plotted with matplotlib.^56^ Subtraction of controls without protein from the melting curves did not affect the estimation of the melting temperature.

### Ubiquitin-prg Reactions

A 2× stock of USP1^G670A,G671A^ or USP1^Δ1Δ2^ was prepared containing 4 μM protein, 50 μM inhibitor, and 1% DMSO in prg reaction buffer (1× PBS supplemented with 0.4 mM TCEP). A 2× stock of Ub-Prg was prepared in prg reaction buffer, with the concentration of Ub-Prg varying depending on the final assay concentration. For reactions on ice, all components apart from inhibitor were prepared on ice. To initiate reaction, the two stocks were mixed 1:1. Reactions were terminated at indicated time points by mixing with an equal volume of 2× NuPAGE LDS buffer (Thermo Fisher) supplemented with 200 mM DTT. SDS-PAGE was performed using Novex 4–12% Tris-glycine gels (Thermo Fisher) or in-house 9% Tris acrylamide gels. Subsequent staining of the gels was performed with Instant-Blue Coomassie stain (Expedeon). Gels were imaged on an Odyssey CLx (LI-COR) using the 700 or 800 nm channel. Densitometric analysis was performed using Image Studio (LI-COR), quantifying each band and assuming similar staining properties between the reacted and unreacted species.

### Limited Proteolysis

Reactions were performed with 10 μM USP1^Δ1Δ2^ and 25 μM inhibitor or DMSO control in 20 mM Tris pH 8, 150 mM NaCl, 5% glycerol, 0.4 mM TCEP, and 0.5% DMSO with 0.005 mg/mL α-chymotrypsin or trypsin. Reactions were terminated at indicated time points by mixing with an equal volume of 2× NuPAGE LDS buffer (Thermo Fisher) supplemented with 200 mM DTT. SDS-PAGE was performed using Novex 4–12% Bis-Tris gels (Thermo Fisher) and subsequent staining of the gels with Instant-Blue Coomassie stain (Expedeon).

PeptideCutter^57^ (https://web.expasy.org/peptide_cutter/) was used to predict sites with >70 and >90% cleavage probabilities for α-chymotrypsin or trypsin, respectively. An AlphaFold^58^ model of USP1^Δ1Δ2^ was generated using ColabFold^59^ with one recycle and no templates or relaxation. ChimeraX was used to measure the solvent-accessible surface area of the backbone atoms for each predicted site; those computed to be >20 Å^2^ were considered as potential cleavage sites.

## Data Availability

The atomic coordinates have been deposited to the PDB under accession codes 9FCI [10.2210/pdb9FCI/pdb] and 9FCJ [10.2210/pdb9FCJ/pdb]. The cryo-EM maps have been deposited to the EMDB under accession codes EMD-50316 [https://www.ebi.ac.uk/emdb/EMD-50316] and EMD-50317 [https://www.ebi.ac.uk/emdb/EMD-50317].

## Supporting Information Available

Molecular formula strings (CSV)
Figures S1–S11, Tables S1 and S2, and HPLC traces for compounds (PDF)

## Abbreviations Used

BRCA: breast cancer gene
CAS9: CRISPR-associated protein 9
CRISPR: clustered regularly interspaced short palindromic repeats
FANCD2: Fanconi anemia group D2 protein
FANCI: Fanconi anemia group I protein
MIR: mobilized by inhibitor region
PARP: poly(ADP-ribose) polymerase
PCNA: proliferating cell nuclear antigen
Prg: propargylamine
RIR: replaced by inhibitor region
TEV: tobacco etch virus
UAF1: USP1-associated factor 1
Ub: ubiquitin
Ub-Prg: ubiquitin-propargylamine
USP1: ubiquitin-specific protease
