# Supplementary material for: Structural and Biochemical Insights into the Mechanism of Action of the Clinical USP1 Inhibitor, KSQ-4279
Source: J Med Chem. 2024 Aug 27;67(17):15557–68. doi: 10.1021/acs.jmedchem.4c01184 (PMC11403619; doi:10.1021/acs.jmedchem.4c01184)
Supplement: Supplementary file 2 — jm4c01184_si_002.pdf [file jm4c01184_si_002.pdf]

Supporting Information

Title:

Structural and biochemical insights on the mechanism of action of the clinical USP1 inhibitor, KSQ-4279

Authors:

Martin Luke Rennie[1]*, Mehmet Gundogdu[2], Connor Arkinson[1], Steven Liness[2], Sheelagh Frame[2], Helen Walden[1]*

Affiliations:

[1]School of Molecular Biosciences, College of Medical Veterinary and Life Sciences, University of Glasgow; Glasgow, UK

[2]Ubiquigent Ltd, Dundee University Incubator, James Lindsay Place, Dundee, UK

*Co-corresponding authors. Martin Luke Rennie, martin.rennie@glasgow.ac.uk; Helen Walden, helen.walden@glasgow.ac.uk

Table of Contents

SUPPLEMENTARY FIGURES AND TABLES

Figure S1.. **S2**
Figure S2.. **S3**
Figure S3.. **S4**
Figure S4.. **S5**
Figure S5.. **S6**
Figure S6.. **S7**
Figure S7.. **S8**
Figure S8.. **S9**
Figure S9.. **S10**
Figure S10.. **S11**
Figure S11.. **S12**
Table S1.. **S13**
Table S2.. **S15**
HPLC traces for compounds.. **S17**

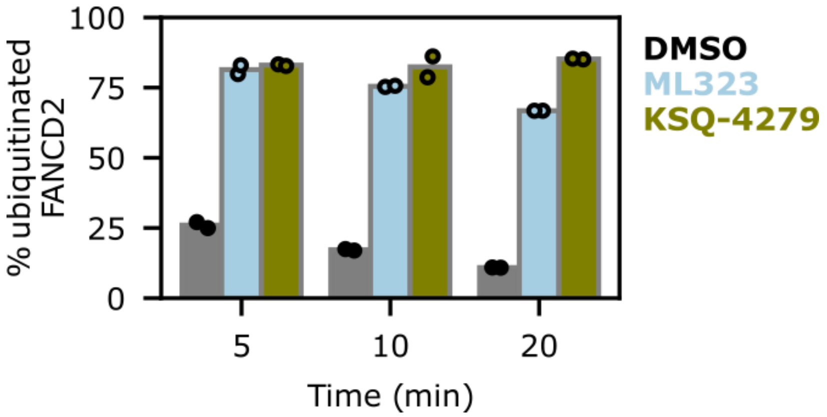

Figure S1: Quantification of coomassie stained gels of FANCI-FANCD2^{Ub} deubiquitination assays. Two technical replicates were performed.

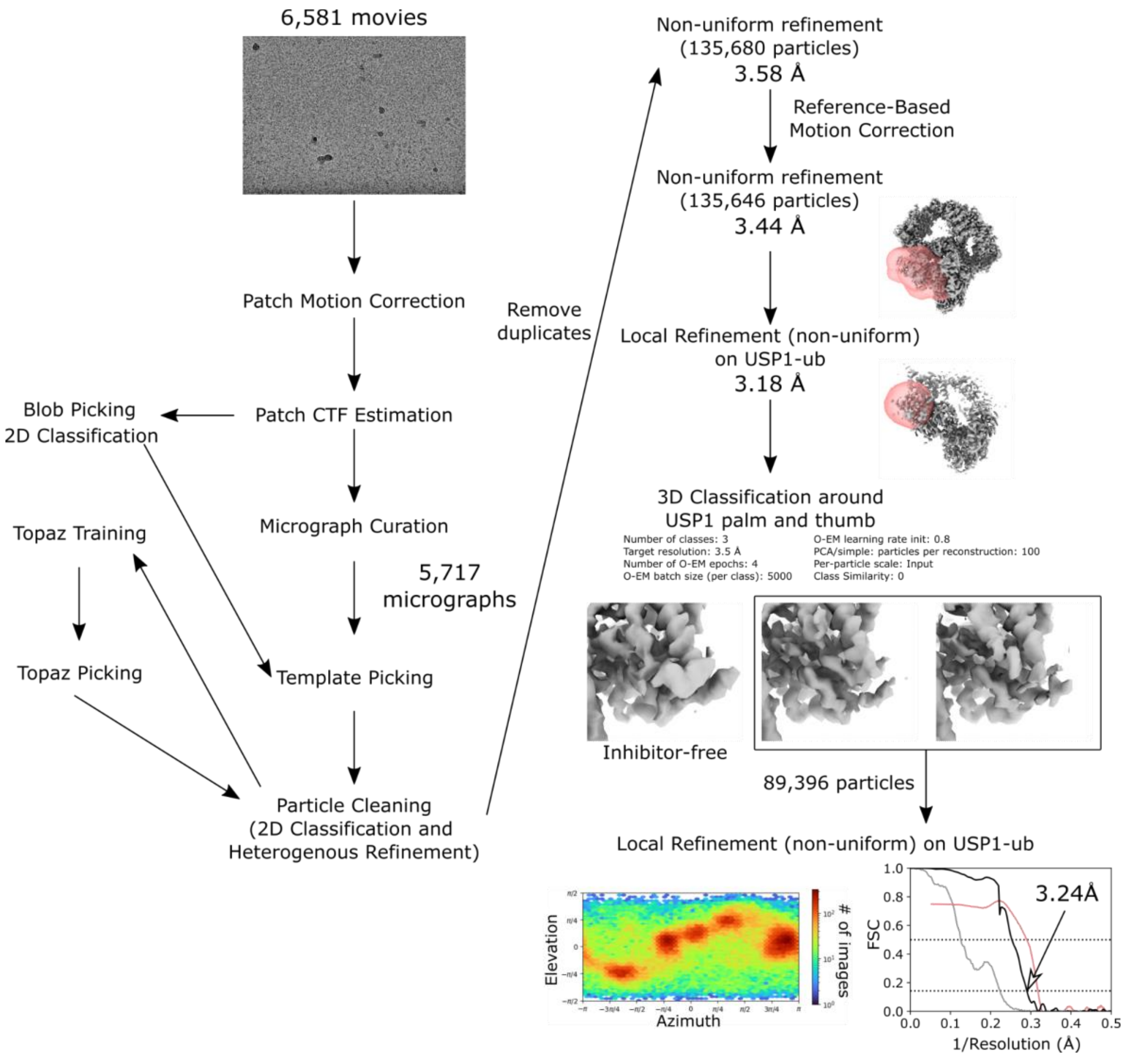

Figure S2: Cryo-EM particle processing workflow for the KSQ-4279-bound structure. Half-map FSCs for no mask (dark gray) and tight mask with correction by noise substitution (black) and distributions of particle orientations are shown for the final reconstruction. Model-map FSC is shown in pink.

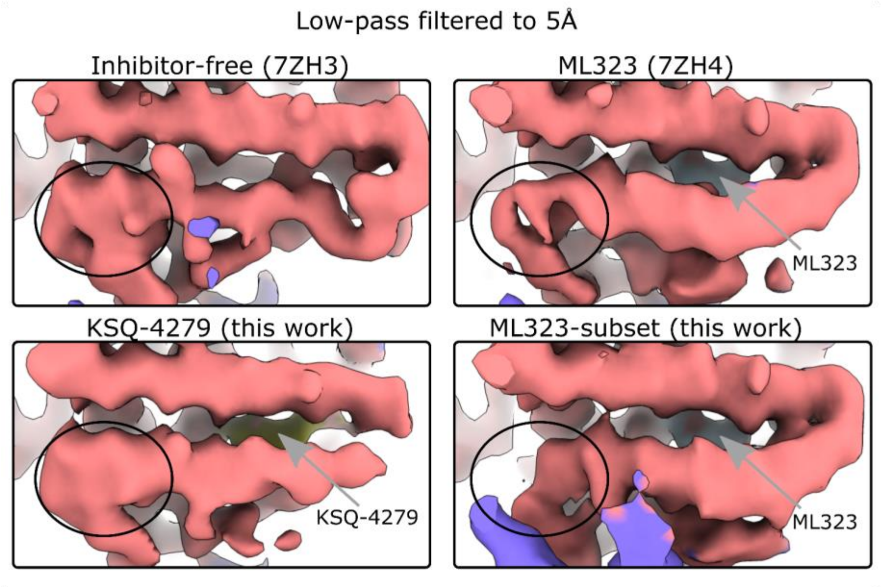

Figure S3: Comparison of the disordered region between ML323 and KSQ-4279 in the cryo-EM maps. Cryo-EM maps were low-pass filtered to 5 Å to mitigate differences in resolution. The circled region is similar between the inhibitor-free and KSQ-4279 structures, while with ML323 there are at least two conformations present.

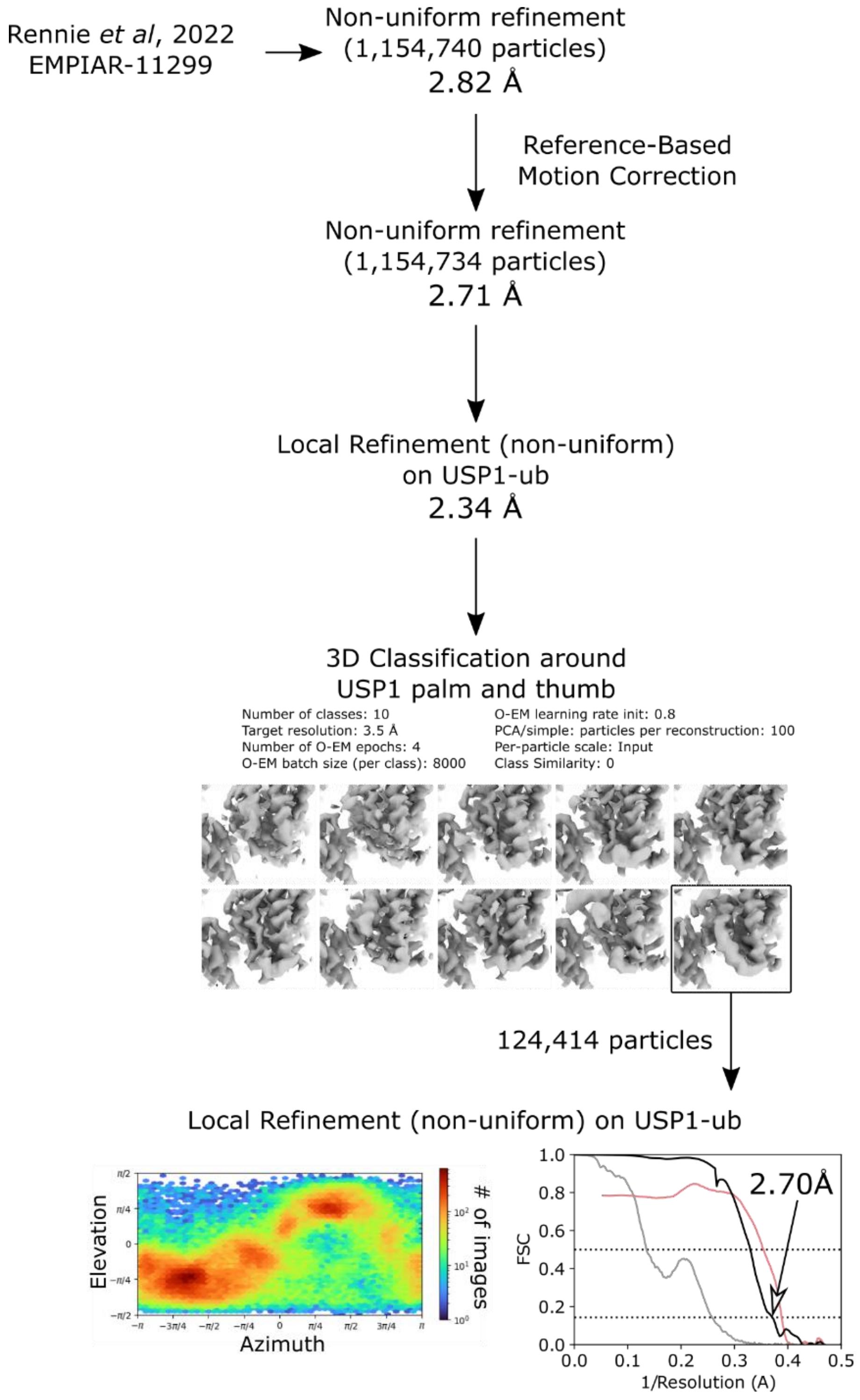

Figure S4: Cryo-EM particle processing workflow for the ML323^{subset} structure. Half-map FSCs for no mask (dark gray) and tight mask with correction by noise substitution (black) and distributions of particle orientations are shown for the final reconstruction. Model-map FSC is shown in pink.

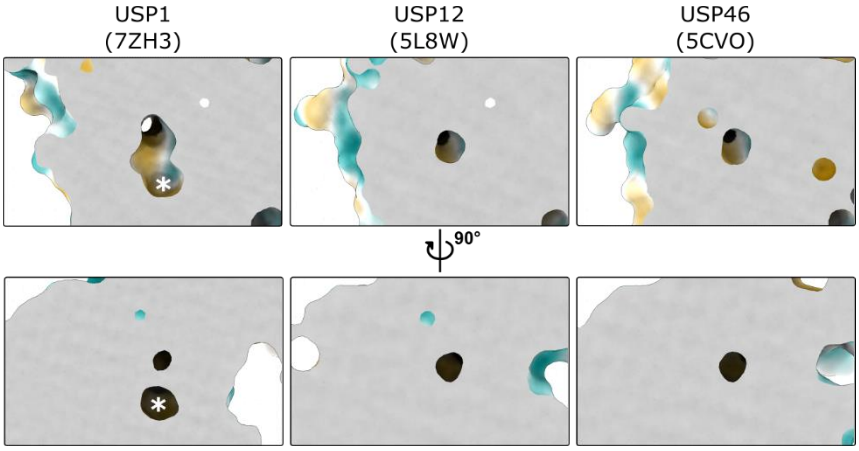

Figure S5: Solvent-excluded surface of inhibitor-free USP1 (7ZH3[29]), USP12 (5L8W[33]), and USP46 (5CVO31). Solvent-excluded surfaces are coloured by lipophilicity and inhibitors as sticks. USP12 and USP46 were superposed onto the USP1 structure (7ZH3[29]).

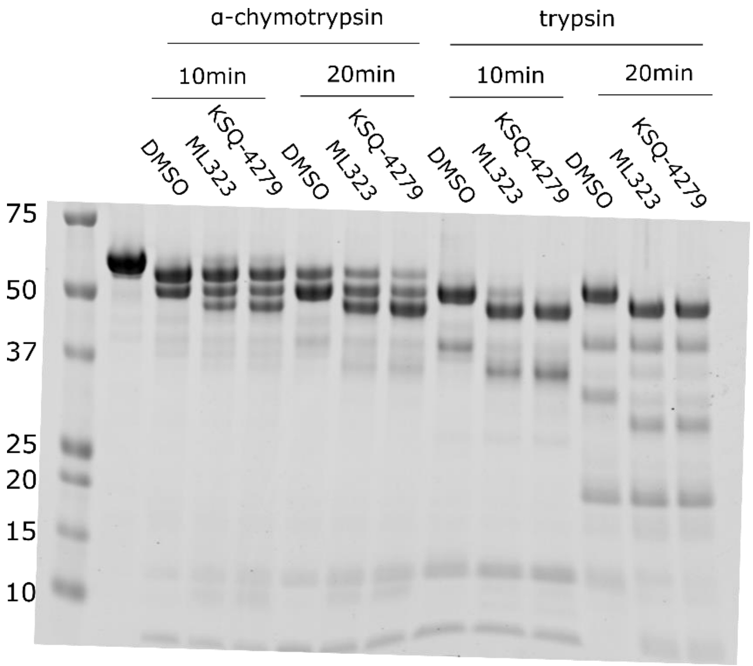

Figure S6: Limited proteolysis of USP1$^{\Delta1\Delta2}$. Uncropped gel from Figure 6D.

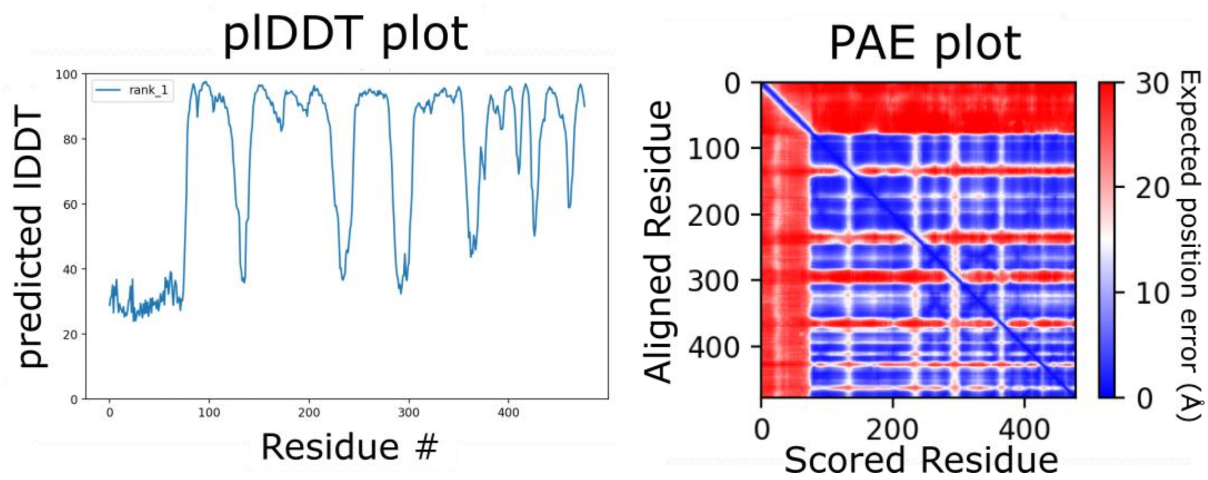

Figure S7: Confidence metrics for the USP1$^{\Delta1\Delta2}$ AlphaFold model.

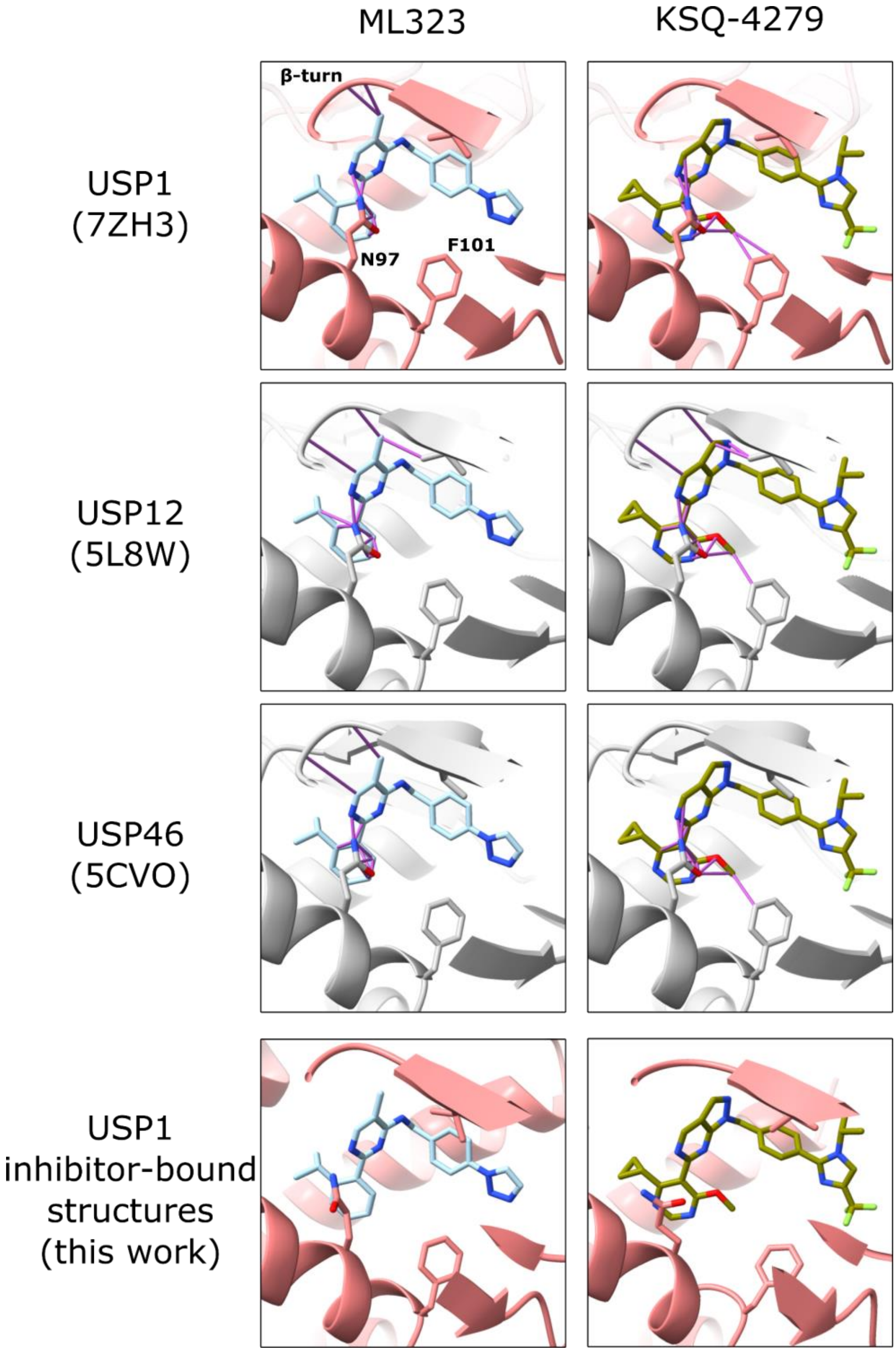

Figure S8: Superposition of the inhibitor-bound structures onto inhibitor-free USP1 (7ZH3[29]), USP12 (5L8W[33]) and USP46 (5CVO[31]) structures. The RIR and MIR are hidden. Clashes between non-RIR or -MIR residues are shown as purple lines.

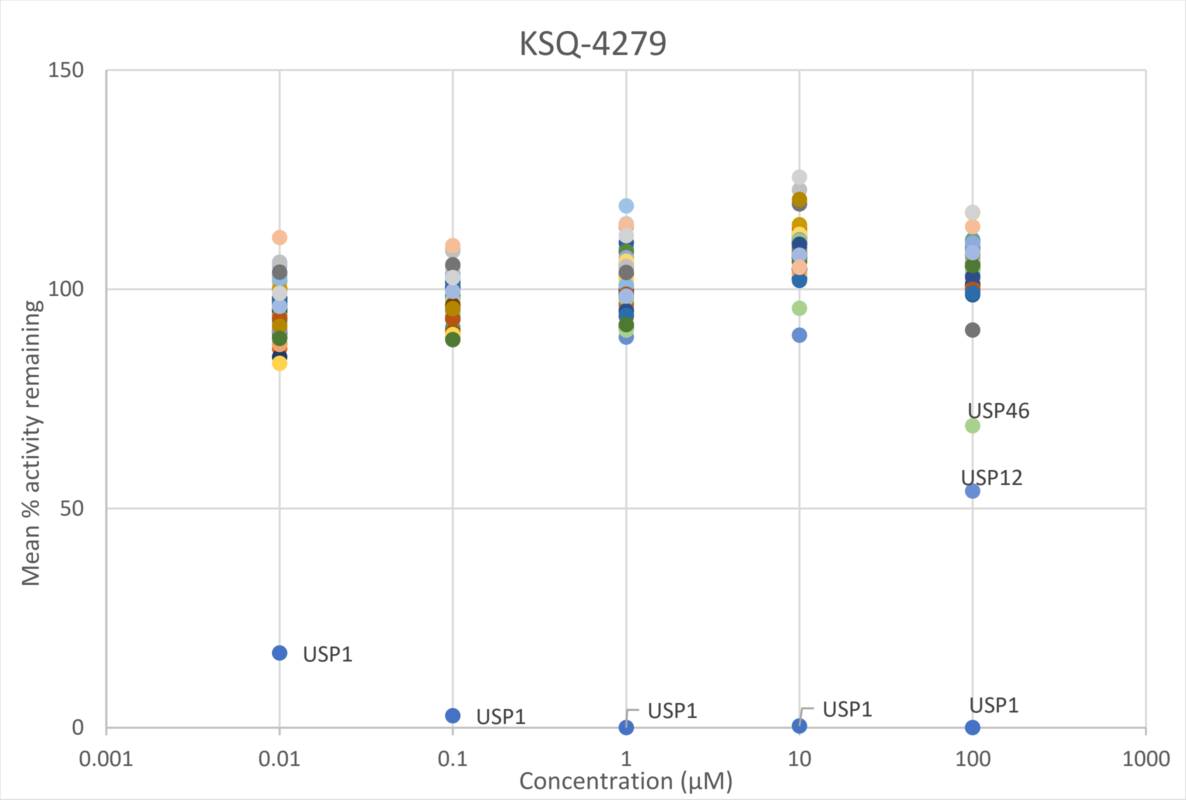

Figure S9: Evaluation of the selectivity of KSQ-4279 compounds across the DUBprofiler™ panel (Ubiquigent).

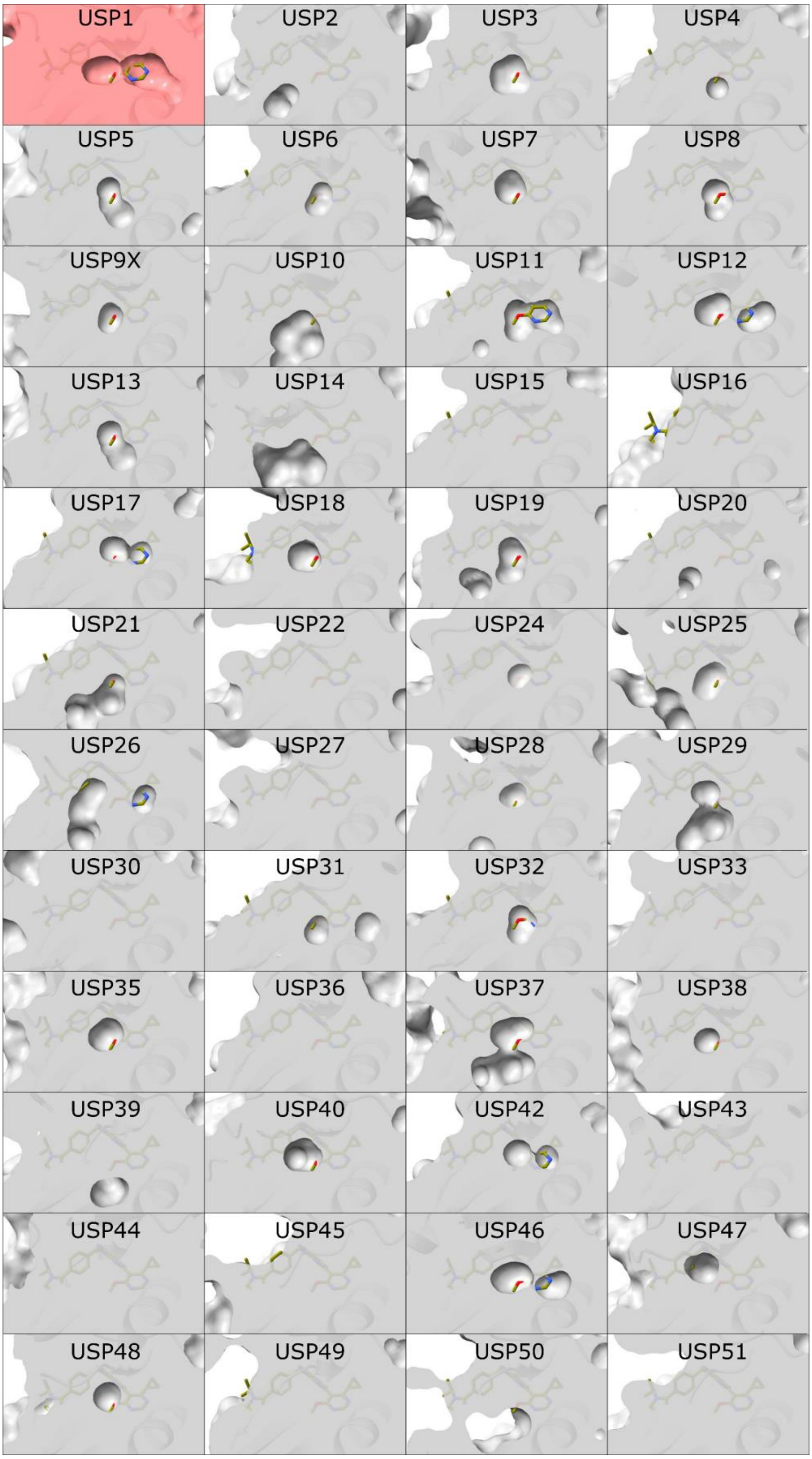

Figure S10: Superposition of the KSQ-4279-bound structure onto AlphaFold models of 48 USPs, focusing on the hydrophobic tunnel.

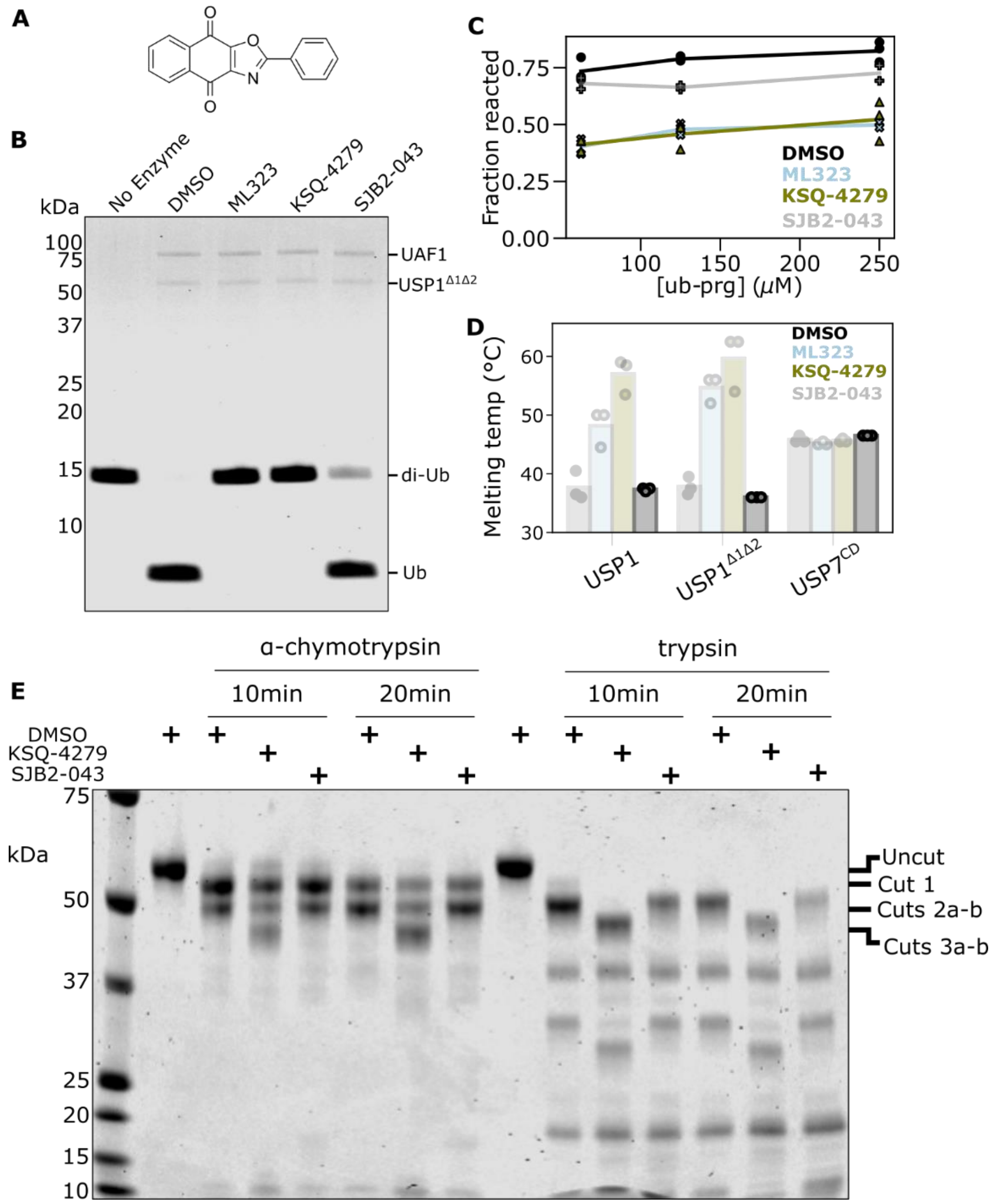

Figure S11: Characterization of SJB2-043 mediated inhibition of USP1. (A) Chemical structure of SJB2-043. (B) Gel-based deubiquitination assays with K48-linked di-ubiquitin substrate in the presence or absence of 25 µM inhibitor. USP1$^{\Delta1\Delta2}$-UAF1 enzyme and di-ubiquitin substrate were used at 0.005 µM and 5 µM, respectively. (C) Gel-based assay of reactions of 2 µM USP1$^{\Delta1\Delta2}$ alone or with 25 µM inhibitor with excess Ub-Prg on ice using a single 3 min time-point. Fraction reacted was quantified using densitometric analysis of the bands. At least two technical replicates were performed. (D) Thermal shift assays with SJB2-043. Data from Figure 5B are shown transparently to facilitate comparison. (E) Limited proteolysis reactions of USP1$^{\Delta1\Delta2}$ with different proteases.

Table S1. Cryo-EM data collection and model refinement statistics

	KSQ-4279 Consensus (before classification)	KSQ-4279 Focused (after classification)	ML323^{subset} Focused (EMPIAR-11299)
**Data collection and processing**			
Microscope	Krios		-
Detector	K3		-
Nominal Magnification	105,000x		-
Voltage (kV)	300		-
Electron Dose ($e^-/Å^2$)	~60		-
Defocus range (μm)	~0.5-2.0		-
Pixel Size (Å)	0.83		1.06
Symmetry imposed	C1		C1
Map resolution (Å)	3.44	3.24	2.70
FSC threshold	0.143	0.143	0.143
Map resolution range (Å)[a]	-	3.1-4.0	2.5-3.5
FSC threshold	-	0.143	0.143
EMDB ID	EMD-50316 (additional map)	EMD-50316	EMD-50317
**Refinement**			
Initial models used	-	7ZH4	7ZH4
Map sharpening B-factor ($Å^2$)	-	97.9	81.5
Correlation coefficient (mask)[b]	-	0.84	0.86
Bond length rmsd (Å)	-	0.004	0.003
Bond angle rmsd (°)	-	0.625	0.539
All-atom clashscore	-	8.82	4.75
Ramachandran plot	-		
Outliers (%)		0.00	0.00
Allowed (%)		2.49	1.60
Favored (%)		97.51	98.40
Rama-Z (whole)	-	-0.85	0.03

CaBLAM Outliers (%)		0.87	0.56
Rotamer outliers (%)		0.00	0.56
PDB ID	-	9FCI	9FCJ

[a]1% and 99% quantiles from local resolution computed in cryoSPARC with Adaptive Window Factor of 20

[b]Calculated in phenix

Table S2. Protein purification buffers.

	Purification step (column)/Experiment	Buffer composition
USP1 or UAF1	Lysis	50 mM Tris pH 8.0, 150 mM NaCl, 5% (v/v) glycerol, 10 mM β-mercaptoethanol, 10 mM Imidazole, 2 mM $MgCl_2$, 1x cOmplete EDTA-free protease inhibitor cocktail, >10 units/mL benzonase
	Ni-NTA Wash 1/Subtractive	50 mM Tris pH 8.0, 500 mM NaCl, 5% (v/v) glycerol, 10 mM β-mercaptoethanol, 10 mM Imidazole
	Ni-NTA Wash 2	50 mM Tris pH 8.0, 100 mM NaCl, 5% (v/v) glycerol, 1 mM TCEP, 10 mM Imidazole
	Ni-NTA Elution	50 mM Tris pH 8.0, 75 mM NaCl, 5% (v/v) glycerol, 1 mM TCEP, 250 mM Imidazole
	Anion Exchange (ResourceQ 1 mL)	50 mM Tris pH 8.0, 5% (v/v) glycerol, 1 mM TCEP, 100-1000 mM NaCl
	Gel Filtration (Superdex 200 Increase 10/300 GL)	20 mM Tris pH 8.0, 150 mM NaCl, 5% (v/v) glycerol, 5 mM DTT
FANCD2, FANCI	Lysis	50 mM Tris pH 8.0, 400 mM NaCl, 5% (v/v) glycerol, 5 mM β-mercaptoethanol, 10 mM Imidazole, 2 mM $MgCl_2$, 1x cOmplete EDTA-free protease inhibitor cocktail, >10 units/mL benzonase
	Ni-NTA Wash 1	50 mM Tris pH 8.0, 400 mM NaCl, 5% (v/v) glycerol, 5 mM β-mercaptoethanol, 10 mM Imidazole
	Ni-NTA Wash 2	50 mM Tris pH 8.0, 150 mM NaCl, 5% (v/v) glycerol, 1 mM TCEP, 10 mM Imidazole
	Ni-NTA Elution	50 mM Tris pH 8.0, 100 mM NaCl, 5% (v/v) glycerol, 1 mM TCEP, 250 mM Imidazole
	Anion Exchange (HP Q 5 mL)	50 mM Tris pH 8.0, 5% (v/v) glycerol, 1 mM TCEP, 100-1000 mM NaCl
	Gel Filtration (Superose 6 Increase 10/300 GL)	20 mM Tris pH 8.0, 400 mM NaCl, 5% (v/v) glycerol, 5 mM DTT
$USP7^{CD}$	Lysis Buffer	50 mM Tris pH 8.0, 150 mM NaCl, 5% (v/v) glycerol, 10 mM imidazole, 10 mM β-ME, 1 mM MgCl2, 1x cOmplete EDTA-free protease inhibitor cocktail, >10 units/mL benzonase
	Wash Buffer 1	50 mM Tris pH 8.0, 500 mM NaCl, 5% (v/v) glycerol, 10 mM imidazole, 10 mM β-ME
	Wash Buffer 2	50 mM Tris pH 8.0, 150 mM NaCl, 5% (v/v) glycerol, 10 mM imidazole, 10 mM β-ME

	Anion Exchange (ResourceQ 1 mL)	20 mM Tris pH 8.0, 5% (v/v) glycerol, 10 μM β-ME, 100-1000 mM NaCl
	Gel Filtration (Superdex75 10/300 GL)	20 mM Tris pH 8.0, 150 mM NaCl, 5% (v/v) glycerol, 5 mM DTT
Ubiquitin-prg	Lysis	20 mM Na_2HPO_4 pH 7.2, 200 mM NaCl, 1 mM EDTA, protease inhibitor cocktail (30mL/1L cell culture)
	Wash Buffer 1	20 mM Na_2HPO_4 pH 7.2, 200 mM NaCl, 0.1 mM EDTA
	Wash Buffer 2	20 mM Na_2HPO_4 pH6.0, 200 mM NaCl, 1 mM EDTA
	Elution Buffer	20 mM Na_2HPO_4 pH6.0, 200 mM NaCl, 1 mM EDTA, 200 mM MESNa
	Gel Filtration (Superdex75 16/600)	1xPBS

HPLC traces for compounds

Data for ML323 Batch #11267 from MedChemExpress

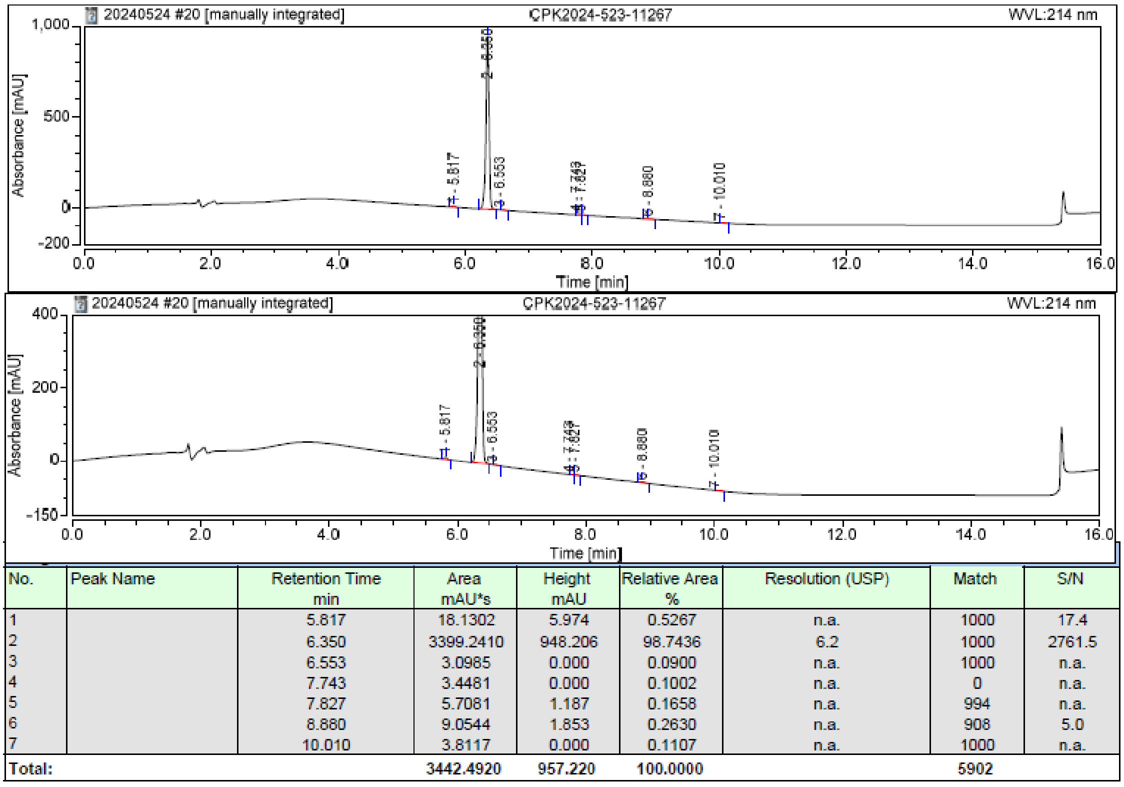

No.	Peak Name	Retention Time min	Area mAU*s	Height mAU	Relative Area %	Resolution (USP)	Match	S/N
1		5.817	18.1302	5.974	0.5267	n.a.	1000	17.4
2		6.350	3399.2410	948.206	98.7436	6.2	1000	2761.5
3		6.553	3.0985	0.000	0.0900	n.a.	1000	n.a.
4		7.743	3.4481	0.000	0.1002	n.a.	0	n.a.
5		7.827	5.7081	1.187	0.1658	n.a.	994	n.a.
6		8.880	9.0544	1.853	0.2630	n.a.	908	5.0
7		10.010	3.8117	0.000	0.1107	n.a.	1000	n.a.
Total:			3442.4920	957.220	100.0000		5902	

Data for KSQ-4279 Batch #180108 from MedChemExpress

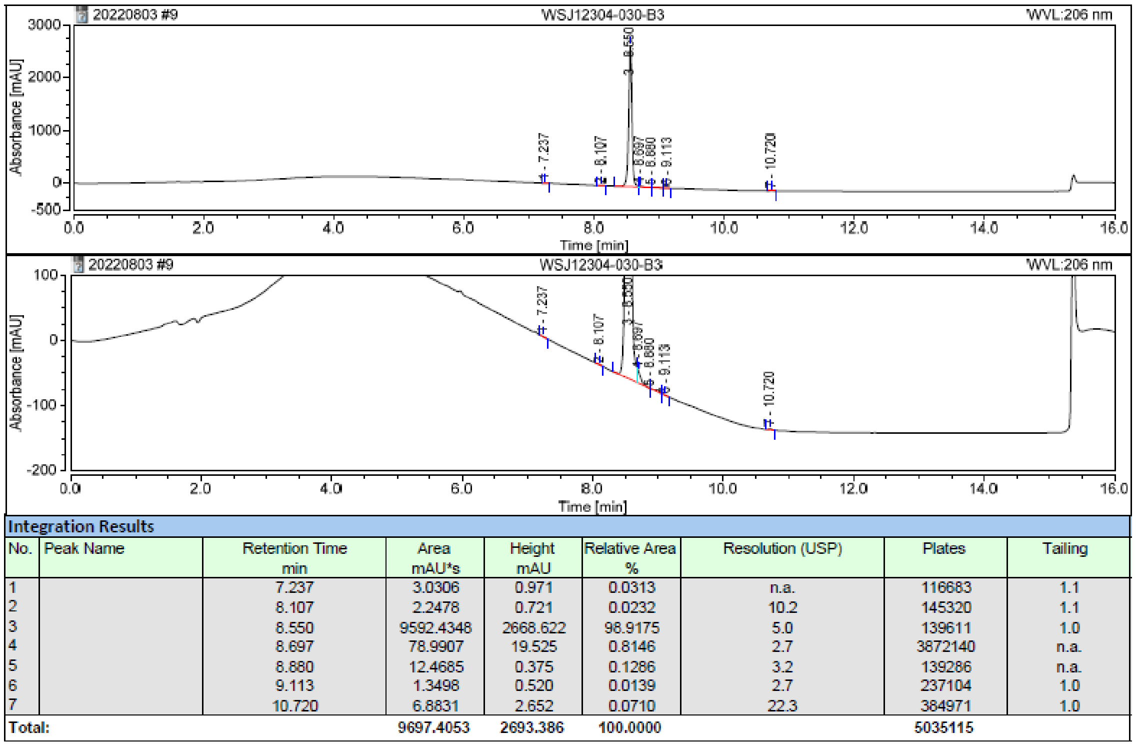

Integration Results

No.	Peak Name	Retention Time min	Area mAU*s	Height mAU	Relative Area %	Resolution (USP)	Plates	Tailing
1		7.237	3.0306	0.971	0.0313	n.a.	116683	1.1
2		8.107	2.2478	0.721	0.0232	10.2	145320	1.1
3		8.550	9592.4348	2668.622	98.9175	5.0	139611	1.0
4		8.697	78.9907	19.525	0.8146	2.7	3872140	n.a.
5		8.880	12.4685	0.375	0.1286	3.2	139286	n.a.
6		9.113	1.3498	0.520	0.0139	2.7	237104	1.0
7		10.720	6.8831	2.652	0.0710	22.3	384971	1.0
Total:			9697.4053	2693.386	100.0000		5035115	
